# Improving stability of prediction models based on correlated omics data by using network approaches

**DOI:** 10.1371/journal.pone.0192853

**Published:** 2018-02-20

**Authors:** Renaud Tissier, Jeanine Houwing-Duistermaat, Mar Rodríguez-Girondo

**Affiliations:** 1 Department of Medical Statistics and Bioinformatics, Leiden University Medical Centre, Leiden, The Netherlands; 2 Developmental and Educational Psychology, Universiteit Leiden Faculteit Sociale Wetenschappen, Leiden, The Netherlands; 3 Department of Statistics, University of Leeds, Leeds, United Kingdom; Universitatsmedizin Greifswald, GERMANY

## Abstract

Building prediction models based on complex omics datasets such as transcriptomics, proteomics, metabolomics remains a challenge in bioinformatics and biostatistics. Regularized regression techniques are typically used to deal with the high dimensionality of these datasets. However, due to the presence of correlation in the datasets, it is difficult to select the best model and application of these methods yields unstable results. We propose a novel strategy for model selection where the obtained models also perform well in terms of overall predictability. Several three step approaches are considered, where the steps are 1) network construction, 2) clustering to empirically derive modules or pathways, and 3) building a prediction model incorporating the information on the modules. For the first step, we use weighted correlation networks and Gaussian graphical modelling. Identification of groups of features is performed by hierarchical clustering. The grouping information is included in the prediction model by using group-based variable selection or group-specific penalization. We compare the performance of our new approaches with standard regularized regression via simulations. Based on these results we provide recommendations for selecting a strategy for building a prediction model given the specific goal of the analysis and the sizes of the datasets. Finally we illustrate the advantages of our approach by application of the methodology to two problems, namely prediction of body mass index in the DIetary, Lifestyle, and Genetic determinants of Obesity and Metabolic syndrome study (DILGOM) and prediction of response of each breast cancer cell line to treatment with specific drugs using a breast cancer cell lines pharmacogenomics dataset.

## Introduction

The advent of the omic era in biomedical research has led to the availability of an increasing number of omics measurements representing various biological levels. Omics datasets (e.g. genomics, methylomics, proteomics, metabolomics, and glycomics) are measured to provide insight in biological mechanisms. In addition, new predictions models can be built based on omics predictors. Omic data are typically high-dimensional (i.e. *n* < *p*, *n* sample size and *p* the number of variables) and they present unknown dependence structures reflecting various biological pathways, co-regulation, biological similarity or coordinated functions of groups of features. Since traditional regression methods have been developed for low-dimensional settings only, they are too restrictive and hence unable to deal with omic datasets and to determine the actual role of their various components. As a result, an important methodological challenge in omic research is how to incorporate these complex datasets in prediction models for health outcomes of interest. This paper is motivated by the previous work of Rodríguez-Girondo [1] in which we showed that metabolomics were predictive of future Body Mass index (BMI) using data from the DIetary, Lifestyle, and Genetic determinants of Obesity and Metabolic syndrome study (DILGOM) [2]. However, when we tried to identify the important metabolites, using lasso regression for variable selection in a cross-validation framework, we obtained inconsistent effect sizes and variable selection frequencies. Specifically, metabolites with largest effects were not always selected and highly correlated variables presented different selection frequencies. These results inspired us to develop more stable prediction models by using network methods.

To obtain a good balance between stability and predictive ability, we propose to incorporate information on the structure between features from an omics dataset into predictions models for health outcomes. The incorporation of such a structure in prediction models is a relatively new and expanding strategy in prediction models. For classification problems methods have been developed, such as the partial correlation coefficient matrix (PPCM) method [3], network-based support vector machines [4], or the selection protein-protein interactions discriminative subnetworks [5]. In this paper we focus on the prediction on continuous outcomes. Also several methods have been developed for this type of outcomes. Zang and Horvath [6], and Reis [7] have proposed to identify clusters of related variables inside the network and to include a summary measure of these clusters, namely principal components and partial least squares. While these approaches provide good results in terms of prediction accuracy, one of their major drawbacks is the chosen summary measures which are hard to interpret and replicate. An alternative approach is network penalization as proposed by Li and Li [8], using the laplacian matrix of the network matrix to build a lasso-type penalization in order to force the effect sizes of variables related to each other in the network to be similar. However, it is relatively heavy in terms of computations and therefore not able to handle too large datasets. Winter *et al.* [9] proposed to first rank variables based on their univariate association with the outcome and their relationships between each other and then use the top ranked variables in a prediction model. While this approach can provide good predictions in some settings, it depends on various tuning parameters and therefore reproducibility is a challenge. Recently, network-based boosting methods [10] and combination of network-based boosting and kernel approaches [11] have been proposed to improve prediction models for GWAs and gene expression studies. These methods include known relationships between genetic markers and phenotypes of interest in order to detect new genetic-phenotypes relationship and therefore improve prediction models. However, for some omic type of data, such as metabolomics and transcriptomics, our lack of knowledge limits the application of these methods only to certain omic sources such as genomics.

**Fig 1:**
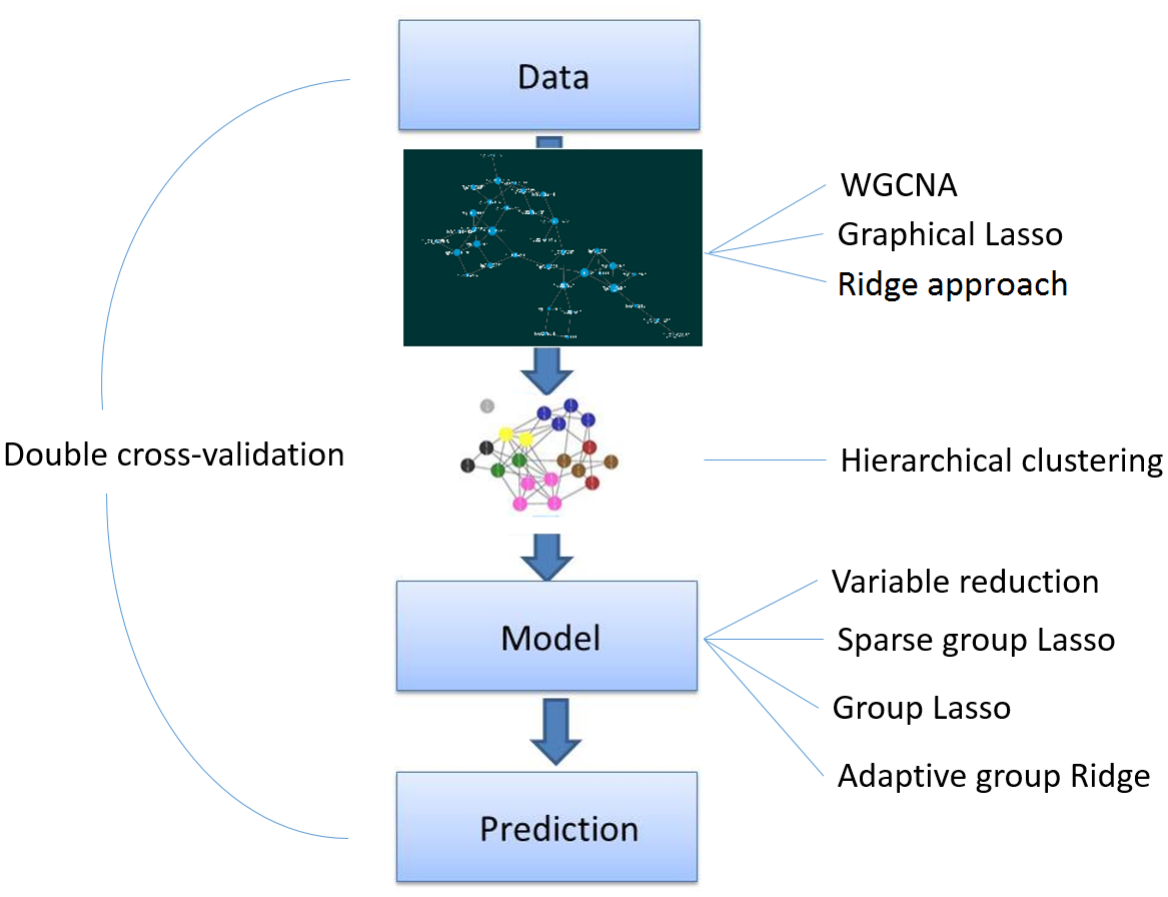
Method summary. Step 1: Networks of features are derived from the data. Step 2: Using hierarchical clustering, modules of features are identified. Step 3: Prediction models are derived using grouping information from Step 2.

In this paper, we propose a flexible approach allowing investigators to apply several types of network analysis approaches to estimate the structure of the data as well as several possible group-penalizations methods. Namely, our approach consists of three steps (Fig 1): network analysis (to empirically derive relations within an omic dataset), clustering (to empirically establish groups of omic related features) and predictive modeling using the aforementioned grouping structure (via group-based variable reduction or group-penalization). This strategy allows a lot of flexibility in terms of both network analysis and prediction models, as different type of omics data have different properties and might need different network analysis strategies or prediction models to obtain proper and biologically relevant results. Finally, to avoid overoptimism in absence of an external validation set, a common situation in omic research, cross-validation of the whole three-step procedure is used.

The rest of the paper is organized as follows: we present the various methods involved in our three-step approach. An intensive simulation study is then presented to empirically evaluate the performance of the various studied methods in terms of predictive ability and variable selection properties. Standard regularized regression methods such as lasso, ridge and elastic net are also considered. The methods are applied to two sets of omic sources (metabolomics and transcriptomics) measured at baseline for the prediction of BMI after seven years of follow-up using DILGOM and on gene expression to predict treatment response from the publicly available breast cancer cell line pharmacogenomics dataset (https://genomeinterpretation.org/content/breast-cancer-cell-line-pharmacogenomics-dataset). In the last section, the results are discussed and concluding remarks are provided.

## Methods

A common approach to build prediction models in high-dimensional settings or in presence of strong correlation between features is regularized regression [12], which has shown to have good properties in terms of predictive ability in various omic settings [13–16]. The choice of the shrinkage type imposes certain constrains in the estimated parameters which can lead to unstability or to models which are difficult to interpret. The lasso approach [17] introduces a *l*_1_-norm constrain of the vector *β* of regression coefficients and shrinks some of the regression coefficients towards zero, introducing sparsity by only selecting ‘the most important variables’ in the model. In the presence of (groups of) correlated features, lasso penalization appears not to perform well in terms of stability since it tends to randomly choose among the strongly correlated features and can select at most *n* variables before saturation. Alternatively, ridge regression [18] considers a *l*_2_-norm constrain of the regression coefficients, which does not allow for explicit variable selection but typically handles well strong correlations. Still, these ridge models are difficult to interpret since sparsity is not obtained. Alternative penalizations as elastic net [19] have been proposed to overcome limitations of lasso and ridge regression, producing sparse models but also allowing to select more than *n* correlated variables.

In the rest of this paper, let the observed data be given by (**y**, **X**), where **y** = (*y*_1_, …, *y*_*n*_)^**T**^ is the continuous outcome measured in *n* independent individuals and **X** is a matrix of dimension *n* × *p*, representing an omic predictor source with *p* features. We propose a three-step approach (Fig 1) to get an interpretable prediction model for **y** based on **X**, where **X** is high-dimensional (*p* > *n*). In the first step, we estimate the intensity matrix (network) of **X**, which contains the degree of relation among the features of **X**. We investigate three different techniques for network estimation: weighted gene co-expression network analysis (WGCNA, [6]), where the relationship is based on Pearson correlation, and two proposals based on gaussian graphical modeling [20], where the relationship is given by the precision matrix. Here two different penalization methods are considered. Namely, ridge [21] and lasso [22]. In the second step, we identify modules (groups) of features by applying hierarchical clustering to the dissimilarity matrix obtained from the estimated network of Step 1. The grouping information is incorporated in the prediction model. Here we consider two strategies: group-based variable reduction and group-penalization. In the variable reduction approach, ‘hubs’ in each group are identified, i.e. variables with the strongest connectivity within a module, and then included in a standard regression. Group penalization, such as adaptive group ridge [23], group lasso [24], and sparse group lasso [25], penalizes the features from the same module jointly. Finally, double cross-validation [1, 26, 27] was applied, over all steps, to obtain proper tuning parameters and summary performance measures in absence of an external validation set.

### Step 1: Network construction

A network is, by definition, an adjacency matrix **A** = [*a*_*ij*_], where *a*_*ij*_ is either an indicator of presence of connection (edge) between two features (nodes) *x*_*i*_ and *x*_*j*_ or a value between 0 and 1 which represents how close the two nodes are. We focus on the latter case because of its continuous nature, and we refer to the resulting networks as weighted networks.

#### WGCNA

Co-expression networks based on pairwise correlations have been proposed in the context of analyzing gene expression data [6]. Due to the presence of many correlated gene expression data, a parameter *β* (soft threshold) is introduced in order to shrink “low” pairwise correlation values towards zero. The parameter *β* might be chosen in such a way that the free-scale topology criterion holds, i.e, the fraction of nodes with *k* edges should follow the power law *P*(*k*) ≈ *k*^−*γ*^, with *P*(*k*) the fraction of nodes in the network with *k* edges and *γ* a constant with a value comprised between 2 and 3. The rationale behind the free scale topology criterion relies on maximizing the within cluster connectivity while minimizing the between cluster connectivity.

Co-expression networks have been successfully used in the context of transcriptomics [28–30]. A drawback of the approach is that the soft thresholding does not provide a sparse network as none of the correlation coefficients is set to zero. In some omic settings, such as metabolomics and glycomics where correlations are high the network might be too dense to interpret. This limitation has motivated the use of alternative approaches such as Gaussian graphical models based on partial correlations which are, by definition, more sparse.

#### Gaussian graphical modeling

Partial correlation coefficients represent the pairwise correlation between two variables conditional on all other variables. Thus the linear effects of all other variables are removed and association is based on the remaining signals. The use of partial correlations appears to provide sparser and more biologically relevant networks compared to networks based on Pearson correlation [31, 32].

In the low-dimensional setting (*p* < *n*) the partial correlation matrix is straightforward estimated as $P=-scale\left(S^{-1}\right)=-diag{\left(S\right)}^{-\frac{1}{2}}Sdiag{\left(S\right)}^{-\frac{1}{2}}$, where **S** is the sample variance-covariance matrix. However, note that the calculation of partial correlations relies on the inversion of the sample variance-covariance matrix, which is challenging (or impossible) in case of strong collinearity between variables or in high-dimensional (*p* > *n*) situations. To overcome this difficulty, several authors have considered penalizing the covariance matrix in order to invert it. In this work, we focus on two methods namely a ridge-type [21] and a lasso-type penalty [22].

**Ridge-penalty approach** Ha and Sun [21] proposed a method to obtain a sparse partial correlation matrix, based on a ridge-type penalty to invert the variance-covariance matrix. Specifically, let **S** be the empirical variance-covariance matrix. To deal with singularity of **S** due to collinearity or high-dimension a positive constant to the diagonal elements of **S** is added, $S^{\prime }=S+\lambda I_{p}$. For any λ > 0, **S**′ has full rank. The partial correlation matrix **R** is estimated as follows:

$$\begin{array}{c} \hat{R}=-scale\left(S^{\prime -1}\right) \end{array}$$

When the penalty parameter λ goes to infinity, the partial correlation matrix is shrunk towards the identity matrix. To obtain a sparse matrix, it is tested whether each coefficient *r*_*ij*_ is significantly different from zero by applying a Fisher’s z-transformation [33] on the partial correlation estimates and assuming that these transformations follow a mixture of null and alternative hypotheses. Efron’s central matching method [34] allows to estimate the null distribution of this test statistic by approximating the mixture distribution using polynomial Poisson regression. Thus, p-values can be computed for each estimated partial correlation *r*_*ij*_, and a sparse network (if *r*_*ij*_ not significant, *r*_*ij*_ is set to zero) is obtained.

**Lasso approach** An alternative penalization method is to apply a lasso-type penalty when estimating the inverse of the estimated variance-covariance matrix [22]. Assume that we have *n* multivariate normal observations of dimension *p*, with mean vector *μ* and variance-covariance matrix **Σ**. To estimate **S** the following penalized log-likelihood has to be maximized:

$$\begin{array}{c} L\left(\Theta \right)=log\left(det\left(\Theta \right)\right)-trace\left(S\Theta \right)-\lambda {\left||\Theta |\right|}_{1} \end{array}$$

with Θ = **Σ**^−1^. The optimal tuning parameter λ is determined by minimizing the AIC (*AIC* = *n* × *tr*(*S*Θ) − *log*(*det*(Θ)) + 2*E*) with *E* the number of non-zero elements in Θ. Note that, especially for small values of the penalty parameter, the resulting partial correlation matrix is not exactly symmetric. Symmetry can be imposed by duplicating one of the estimated triangular matrices (upper or lower).

### Step 2: Hierarchical clustering

Hierarchical clustering is used to detect groups of related features from the estimated network which was obtained with the methods introduced in the previous section.

Specifically, we have used the dynamic tree cut algorithm based on the dendogram obtained by hierarchical clustering [35]. This is an adaptive and iterative process of cluster decomposition and combination until the number of clusters becomes stable. This approach, in contrast to a constant height cut-off method, is capable of identifying nested clusters and is implemented in the R package WGCNA. The measure used for the hierarchical clustering aproach was the topological overlap dissimilarity measure. The topological overlap of two nodes quantifies their similarity in terms of the commonality of the nodes they connect [36] and is given by:

$$\begin{array}{c} TOM_{ij}=\frac{\sum_{u}a_{iu}a_{uj}+a_{ij}}{min\left(k_{i},k_{j}\right)+1-a_{ij}} \end{array}$$

with *a*_*ij*_ the weight between *i* and *j* in the adjacency matrix, and *k*_*i*_ = ∑_*u*_ *a*_*iu*_. The topological overlap dissimilarity measure is now defined as: *dissTOM*_*ij*_ = 1 − *TOM*_*ij*_.

### Step 3: Outcome prediction

Finally, we incorporate the obtained grouping information in the prediction models. One of the major challenges in prediction using high dimensional data is to avoid overfitting. Overfitting occurs when a model is too complex, i.e when it has too many parameters. We used two of the most standard approaches for parameter reduction which are a priori variable reduction based on variable importance and shrinkage methods. Namely, we consider within-group variable selection and regularized regression models with group penalization. In general, regularized regression models are characterized by the optimization problem $\min_{\beta \in R^{p}}{(∥y-\sum X\beta ∥}_{2}^{2}+R\left(\beta \right))$ where *R*(*β*) is the regularization or penalty term. Examples of commonly used penalization functions are: *R*(*β*) = λ∑_*j*_|*β*_*j*_| (lasso; [17]), $R\left(\beta \right)=\lambda \sum_{j}\beta_{j}^{2}$ (ridge; [18]) and $R\left(\beta \right)=\alpha \sum_{j}\beta_{j}^{2}+\left(1-\alpha \right)\sum_{j}\left|\beta_{j}\right|$ *α* ∈ (0, 1) (elastic net; [19]).

#### Variable importance

The general idea of this simple approach is to retain the most relevant (according to some pre-defined criterion) variables from each of the estimated groups obtained by hierarchical clustering in step 2. We propose to consider only the most strongly connected variables within its group (‘hubs’), assuming that strong connectivity is indicative of biological importance and hence relevance to predict the outcome of interest. Specifically, for a specific group G:

$$\begin{array}{c} hub_{G}=\max_{i}\left(\sum_{j\in G}I_{a_{ij}\neq 0}\right) \end{array}$$

with *a*_*ij*_ the *ij* element of the adjacency matrix. If multiple nodes have the same maximum, all these hubs are selected. Ridge regression is used to deal with collinearity in case of several selected hubs.

#### Group penalization

An alternative to within-cluster variable selection is to consider cluster-based penalties in the context of regularized regression.

**Group lasso** Group lasso [24] selects groups of variables since it simultaneously shrinks all the coefficients belonging to the same group towards zero. The group lasso estimator is given by:

$$\begin{array}{c} \min_{\beta \in R^{p}}(∥y-\sum_{l=1}^{L}X_{l}\beta_{l}{∥}_{2}^{2}+\lambda \sum_{l=1}^{L}\sqrt{p_{l}}∥\beta_{l}{∥}_{2}) \end{array}$$

where *l* ∈ (1⋯*L*) represents the index of the group of predictors, *L* is the the total number of clusters, *X*_*l*_ is the matrix of predictors in the group *l* and $\sqrt{p_{l}}$ is a penalty to take into account the varying group size. The tuning parameter λ is made by cross-validation based on minimization of the AIC. The group lasso estimator is asymptotically consistent even when model complexity increases. Note that if each group contains just one variable, group lasso is equivalent to the standard lasso [17].

**Sparse group lasso** Sparse group lasso [25] can be applied when one also wish to select variables within a group. Shrinkage is carried out at the group level and at the level of the individual features, resulting in the selection of important groups as well as members of those groups. The sparse group lasso estimator is given by:

$$\begin{array}{c} \min_{\beta \in R^{p}}(∥y-\sum_{l=1}^{L}X_{l}\beta_{l}{∥}_{2}^{2}+\left(1-\alpha \right)\lambda \sum_{l=1}^{L}\sqrt{p_{l}}∥\beta_{l}{∥}_{2}+{\alpha \lambda ∥\beta ∥}_{1}) \end{array}$$

where *l*, *X*_*l*_, $\sqrt{p_{l}}$ and are defined as in group lasso. Note that the sparse group lasso is a combination of group lasso and lasso. The parameter *α* regulates the weight of each approach. For *α* = 1 sparse group lasso equals lasso and for *α* = 0 group lasso.

**Adaptive group-regularized ridge regression** Finally, the recently proposed adaptive group ridge approach [23] which extends ridge regularized regression to group penalization is considered. The adaptive group ridge considers group specific penalties λ_*l*_ for the *L* groups. The adaptive group ridge estimator is given by:

$$\begin{array}{c} \min_{\beta \in R^{p}}(∥y-\sum_{l=1}^{L}X_{l}\beta_{l}{∥}_{2}^{2}+\sum_{l=1}^{L}\lambda_{l}\sum_{q\in G_{l}}\beta_{q}^{2}) \end{array}$$

where *l* and *X*_*l*_ are defined as in group lasso, *G*_*l*_ is the lth group of variables and λ_*l*_ is the penalty term for the group *G*_*l*_. The penalty terms can be expressed as: $\lambda_{l}=\lambda_{l}^{\prime }\lambda$ with λ a unique penalty term and $\lambda_{l}^{\prime }$ as penalty multipliers for each group.

### Software implementation

The proposed three-step approach has been implemented in the R function PredNet which is available at github (https://github.com/RenTissier/NetPred). The function allows to apply all the possible combinations of the previously presented network analysis and group penalization methods. The function calls the packages WGCNA (co-expression based on pairwise correlation), huge (gaussian graphical modeling), GGMridge (ridge-penalty approach), grpreg (group lasso), SGL (sparse group lasso), and GRridge (adaptive group-regularized ridge regression).

## Simulation study

### Simulation setup

An intensive simulation study was conducted to study the performance of our proposed prediction methods using estimated grouping information and to compare them wit9h existing regularized regression methods (without grouping information), such as lasso, ridge and elastic net (*α* = 0.5). We also included the special case of ‘known clustering’, in which we assume that the true underlying grouping structure is known, mimicking the situation in which information on biological clustering is available from previous analyses or open source pathway databases. The omic predictor **X** is simulated from a zero-mean multivariate normal distribution with correlation matrix **Σ**. Following the recent literature on pathway and network analysis of omics data [6], we generated **Σ** according to a hub observation model with added realistic noise [37].

The continuous outcome **y** is generated by y=Xβ+ϵ, where *β* is the vector of regression coefficient of size *p*, and *ϵ* ∼ *N*(0, 1). The singular value decomposition (svd; [38]) of **X**, **X** = **U** **D** **U**^*t*^ allows to generate **y** in terms of the various latent modules present in **X** since they represent different independent subspaces of features accounting for different proportions of variation in **X**. In practice, we first generate β*, the regression coefficients corresponding to each independent module (given by **U**), and we then transform it to the predictor space by using $\beta =U^{t}\beta^{*}$.

Within this general framework, we consider three different scenarios: (Scenario a) $\beta_{j}^{*}=0.01$, *j* = 1; $\beta_{j}^{*}=0$, *j* ≠ 1. **y** is then associated to a high variance subspace of **U**, corresponding to the largest eigenvalue of **X**. (Scenario b) $\beta_{j}^{*}=0.01$, *j* = 4; $\beta_{j}^{*}=0$, *j* ≠ 4. The association with **y** relies on a low-variance subspace of **U**. Hence, we expect lower predictive ability of **X** compared to Scenario a. (Scenario c) $\beta_{j}^{*}=0.01$, *j* = 1, 4; $\beta_{j}^{*}=0$, *j* ≠ 1, 4. The association with **y** relies on several subspaces of **U**. As a result, Scenario c is less sparse than Scenarios a and b.

**Fig 2:**
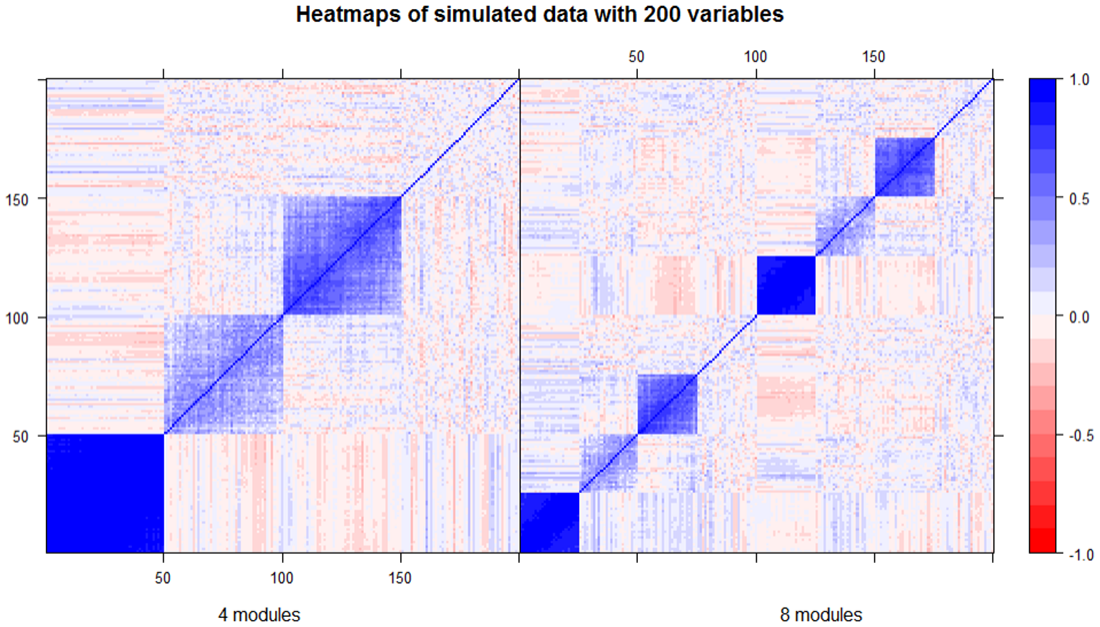
Simulation study; correlation matrices. Example of simulated correlation matrices obtained with 200 variables for 4 and 8 modules respectively.

For each scenario, we considered two sample sizes (*n* = 50 and *n* = 100), different number of features in **X**, (*p* = 200 features and *p* = 4000), and different number of underlying modules (*k* = 4 and *k* = 8). Each module presents various within-correlation levels and in all the scenarios, we assumed the presence of one module of uncorrelated variables. Fig 2 shows the corresponding heatmaps of *Σ* for *k* = 4 (left panel) and *k* = 8 (right panel). For each scenario, we generated *M* = 500 replicates and for each trial we consider 10-fold partitions in order to obtain cross-validated summary measures.

We evaluated our methods in terms of obtaining the correct grouping structure, of prediction performance, and variable selection. Grouping is summarized in two ways. On the one hand, we compared the estimated number of groups with the underlying parameter *k*. On the other hand, for each of the *k* underlying modules, we calculated the correct and incorrect classification rates (belonging or not belonging to the underlying module taken as reference) of each of the *p* features. Predictive ability is measured by $Q^{2}=\frac{\sum_{i=1}^{n}{\left(p_{i}-p_{0i}\right)}^{2}}{\sum_{i=1}^{n}{\left(y_{i}-p_{i}\right)}^{2}}$, the cross-validated version of the fraction of variance explained by the prediction model, in which the performance of the model-based is compared to the naive double cross-validated predictions **p**_**0**_ based on the mean value of the outcome variable **y** [1]. Variable selection properties are assessed by comparing the simulated *β* coefficients with the average estimated regression coefficients.

### Simulation results

**Network analysis and clustering** Tables 1 and 2 show the performance of the studied methods for network analysis and hierarchical clustering. WGCNA obtains number of clusters closer to the truth than graphical lasso and the ridge-penalty approach. WGCNA estimates, on average, $\hat{k}=3$ and $\hat{k}=5$ for *k* = 4 and *k* = 8 underlying modules, respectively. This slight underestimation of *k* yields a large number of false positives (see Table 2). Focusing on the situation of *k* = 4, and taking the group with highest simulated within correlation as reference, Table 2 shows a false positive rate of 38.2% for WGCNA, mainly due to the incorrect assignment of features of the second cluster to the first one. In contrast, graphical lasso overestimates the number of simulated modules.

**Table 1:** Simulation study. Average number of clusters obtained accross cross-validation by WGCNA, graphical lasso, and ridge penalty. The minimum and maximum number of clusters identified are presented in brackets.

	**200 variables**			
	**4 modules**		**8 modules**	
	**n = 50**	**n = 100**	**n = 50**	**n = 100**
WGCNA	3.1(2-5)	3.0(2-5)	5.0(3-8)	5.0(4-7)
Graphical lasso	14.7(9-21)	17.0(12-23)	14.4(9-21)	17.6(13-25)
Ridge penalty	1.0(1-3)	1.3(1-6)	1.5(1-8)	9.8(1-21)
	**1000 variables**			
	**4 modules**		**8 modules**	
	**n = 50**	**n = 100**	**n = 50**	**n = 100**
WGCNA	3.1(2-5)	3.0(2-5)	5.6(4-18)	5.0(4-11)
Graphical lasso	48.3(40-86)	76.5(57-93)	59.6(39-81)	77.5(63-95)
Ridge penalty	10.2(1-71)	52.6(3-72)	13.1(1-69)	61.5(6-81)

**Table 2:** Simulation study. Average (across 10 cross-validation folds and 500 replicates) true positive rate (TPR), false negatives rate (FNR) and false positives rate (FPR) for WGCNA, graphical lasso and ridge penalization. Top part: Scenario a. Reference module: module 1 (corresponding to the first 50 variables in Fig 2 left panel which present the highest level of correlation). Bottom part: Scenario b. Reference module: module 3 (corresponding to the variables 100-150 in Fig 2 left panel).

		50 Individuals			100 Individuals		
		TPR	FNR	FPR	TPR	FNR	FPR
module 1	WGCNA	.999	.001	.382	.998	.002	.375
	Graphical lasso	.308	.692	.000	.259	.741	.000
	Ridge penalty	.999	0.001	.997	.962	.038	.951
module 3	WGCNA	.918	.082	.190	.989	.011	.148
	Graphical lasso	.189	.811	.001	.192	.808	.000
	Ridge penalty	.999	.000	.997	.960	.040	.951

The number of estimated modules is not affected by the number of underlying modules (for example, $\hat{k}=14$ for both *k* = 4 and *k* = 8 with *n* = 50), but it increases with the number of *p* simulated features. This is likely due to the reliance of graphical lasso on partial correlations instead of Pearson correlations. After having a closer look at the estimated modules, we observe that graphical lasso generates $\hat{k}$ groups, which are subsets of the underlying simulated *k* modules. In other words, graphical lasso does not group together features belonging to different underlying modules (WGCNA does), and the estimated modules can be grouped in such a way that the original *k* modules are recovered. This translates in a very small false positive rate when taking any of the *k* simulated modules as reference (see Table 2). Finally, the ridge-penalty approach is, in most of the cases, not able to lead to the identification of any cluster with small number of features and subjects (see *p* = 200 and *n* = 50 in Table 1). For larger number of individuals and variables, the number of clusters is overestimated for the same reason as graphical lasso. Namely, the reliance of this method on partial correlations.

#### Predictive ability

Tables 3 and 4 show the results in terms of the predictive accuracy measure *Q*^2^ for *p* = 200 and *n* = 50 and, for *p* = 1000 and *n* = 50 respectively. Table A and Table B in S1 File, show results for *n* = 100. Adaptive group ridge and group lasso present similar performances in most of the studied situations. These two methods outperform the other considered three-step approaches. Also they are the best performing methods when the known grouping was used. Further, these approaches may outperform the commonly used regularized regression methods lasso, ridge and elastic net regression in terms of predictive ability. Specifically, group lasso relying on grouping structure coming from WGCNA and graphical lasso systematically outperforms ridge and lasso and it presents a similar predictive ability than elastic net when *p* = 200. For *p* = 1000 the predictive ability of the standard ridge, lasso and elastic net is lower while the methods based on group lasso and adaptive group ridge present similar behavior than for *p* = 200. Therefore, the gain of these new approaches appears to be larger when the number of predictors increases.

**Table 3:** Simulation study. Results obtained in terms of average *Q*^2^ (across 500 replicates) for scenarios a, b, c, p = 200 variables, k = 4 and k = 8 modules, and n = 50 individuals. Standard errors are given in brackets. The first column represents the method used to build the network. A Priori represents the situation were the true clustering of the predictors is known and no network analysis is performed.

		4 modules			8 modules		
Scenario		a	b	c	a	b	c
A Priori	Sparse group lasso_0.5_	.79(.01)	.51(.06)	.65(0.02)	.75(.02)	.71(.02)	.69(0.03)
	Sparse group lasso_0.9_	.79(.01)	.48(.06)	.59(.03)	.74(.02)	.69(.04)	.65(0.04)
	Sparse group lasso_0.1_	.79(.01)	.53(.06)	.66(.02)	.75(.02)	.72(.02)	.70(0.03)
	Group lasso	.87(.01)	.53(.07)	.77(.02)	.84(.02)	.78(.03)	.81(0.02)
	Group ridge	.94(.01)	.43(.08)	.69(.07)	.90(.02)	.73(.06)	.85(0.03)
WGCNA	Hubs	.81(.03)	.15(.10)	.59(.11)	.81(.05)	.18(.13)	.55(.12)
	Sparse group lasso_0.5_	.72(.12)	.15(.12)	.57(.15)	.41(.21)	.28(.19)	.36(.20)
	Sparse group lasso_0.9_	.73(.13)	.13(.22)	.53(.13)	.41(.22)	.26(.12)	.35(.19)
	Sparse group lasso_0.1_	.69(.12)	.16(.12)	.58(.15)	.39(.20)	.29(.17)	.36(.19)
	Group Lasso	.90(.02)	.58(.07)	.87(.02)	.83(.04)	.76(.06)	.83(.04)
	Group ridge	.78(.03)	.46(.06)	.62(.05)	.69(.07)	.61(.08)	.53(.09)
Graphical lasso	Hubs	.52(.20)	.26(.15)	.51(.18)	.52(.22)	.45(.20)	.51(.22)
	Sparse group lasso_0.5_	.69(.13)	.08(.06)	.45(.16)	.31(.21)	.22(.15)	.27(.18)
	Sparse group lasso_0.9_	.68(.13)	.06(.05)	.42(.16)	.32(.21)	.19(.15)	.26(.17)
	Sparse group lasso_0.1_	.69(.13)	.08(.06)	.46(.16)	.31(.21)	.24(.15)	.28(.18)
	Group lasso	.92(.01)	.54(.08)	.87(.03)	.86(.03)	.76(.06)	.86(.03)
	Group ridge	.93(.02)	.46(.08)	.61(.06)	.85(.08)	.71(.06)	.70(.11)
Ridge penalty	Hubs	.52(.06)	.11(.02)	.47(.06)	.27(.10)	.22(.07)	.27(.09)
	Sparse group lasso_0.5_	.77(.09)	.42(.05)	.67(.02)	.68(.07)	.63(.04)	.67(.04)
	Sparse group lasso_0.9_	.79(.07)	.46(.06)	.61(.03)	.72(.05)	.66(.05)	.65(.05)
	Sparse group lasso_0.1_	.73(.08)	.40(.04)	.68(.02)	.62(.09)	.59(.03)	.63(.04)
	Group lasso	.87(.02)	.48(.06)	.84(.02)	.79(.04)	.71(.05)	.78(.03)
	Group ridge	.67(.05)	.07(.03)	.69(.05)	.47(.06)	.32(.07)	.45(.07)
Common	Lasso	.88(.03)	.52(.10)	.73(.05)	.81(.04)	.74(0.06)	.79(0.05)
	Ridge	.67(.05)	.07(.03)	.59(.06)	.46(.06)	.55(0.04)	.70(0.03)
	Elastic net	.96(.04)	.74(.26)	.79(.20)	.87(.02)	.81(.04)	.89(.02)

**Table 4:** Simulation study. Results obtained in terms of average *Q*^2^ (across 500 replicates) for scenarios a, b, c, p = 1000 variables, k = 4 and k = 8 modules, and n = 50 individuals. Standard errors are given in brackets. The first column represents the method used to build the network. A Priori represents the situation were the true clustering of the predictors is known and no network analysis is performed.

		4 modules			8 modules		
Scenario		a	b	c	a	b	c
A Priori	Sparse group lasso_0.5_	.80(.002)	.64(.02)	.63(.03)	.77(.016)	.69(.036)	.69(.030)
	Sparse group lasso_0.9_	.80(.001)	.56(.036)	.54(.047)	.76(.019)	.62(.047)	.67(.056)
	Sparse group lasso_0.1_	.80(.002)	.66(.026)	.66(.032)	.77(.016)	.70(.033)	.72(.025)
	Group lasso	.89(.003)	.76(.021)	.71(.046)	.87(.011)	.81(.022)	.84(.016)
	Group ridge	.97(.011)	.65(.076)	.55(.083)	.95(.018)	.87(.033)	.78(.065)
WGCNA	Hubs	.87(.026)	.48(.12)	.45(.324)	.45(.324)	.13(.127)	.08(.088)
	Sparse group lasso_0.5_	.74(.143)	.61(.098)	.57(.153)	.43(.244)	.36(.206)	.32(.221)
	Sparse group lasso_0.9_	.74(.147)	.54(.090)	.53(.138)	.44(.252)	.35(.193)	.29(.223)
	Sparse group lasso_0.1_	.70(.134)	.62(.098)	.58(.155)	.40(.227)	.34(.196)	.32(.205)
	Group lasso	.94(.01)	.85(.031)	.87(.027)	.88(.036)	.79(.043)	.78(.058)
	Group ridge	.80(.037)	.59(.061)	.62(.059)	.70(.067)	.50(.088)	.62(.096)
Graphical lasso	Hubs	.52(.054)	.55(.054)	.21(.039)	.42(.059)	.46(.063)	.43(.050)
	Sparse group lasso_0.5_	.79(.032)	.54(.110)	.12(.08)	.46(.251)	.32(.202)	.34(.185)
	Sparse group lasso_0.9_	.79(.030)	.49(.122)	.09(.075)	.46(.249)	.30(.191)	.30(.195)
	Sparse group lasso_0.1_	.79(.030)	.56(.111)	.13(.083)	.46(.254)	.32(.208)	.37(.180)
	Group lasso	.96(.01)	.81(.039)	.61(.084)	.93(.023)	.83(.044)	.82(.054)
	Group ridge	.96(.02)	.61(.062)	.59(.075)	.81(.127)	.66(.106)	.75(.069)
Ridge penalty	Hubs	.02(.052)	.07(.064)	.01(.028)	.04(.069)	.05(.075)	.05(.060)
	Sparse group lasso_0.5_	.59(.245)	.57(.163)	.13(.14)	.69(.137)	.62(.140)	.59(.136)
	Sparse group lasso_0.9_	.70(.186)	.49(.148)	.13(.149)	.72(.132)	.59(.136)	.60(.147)
	Sparse group lasso_0.1_	.47(.254)	.59(.164)	.13(.127)	.59(.139)	.58(.130)	.53(.116)
	Group lasso	.91(.031)	.79(.029)	.42(.065)	.82(.053)	.75(.042)	.70(.059)
	Group ridge	.75(.07)	.63(.078)	.10(.055)	.53(.097)	.48(.11)	.37(.10)
Common	Lasso	.91(.016)	.59(.060)	.51(.080)	.87(.035)	.68(.065)	.70(.074)
	Ridge	.80(.028)	.73(.037)	.26(.046)	.66(.041)	.63(.044)	.539(.050)
	Elastic net	.92(.015)	.54(.089)	.60(.057)	.87(.032)	.69(.067)	.68(.065)

Compared to adaptive group ridge, group lasso was less sensitive to the chosen network method. Namely, all scenarios adaptive group ridge presents bad performance when using the ridge penalty approach [21] for network construction. The performance of group lasso is robust with respect to the studied network construction methods in all the studied scenarios, and close to its performance when using the true underlying grouping structure. Sparse group lasso provides proper results in terms of prediction ability when the clustering is known a priori, with *Q*^2^ values only slightly lower than the corresponding values of adaptive group ridge and group lasso. However, when the grouping is estimated, its performance drops. The predictive ability appears to drop to a *Q*^2^ < 0.1 for scenario b, which is 8 times lower than the predictive ability obtained with a combination of graphical lasso and group lasso. The variable selection approach based on selecting hubs only provides satisfactory results when using the WGCNA method for network construction in scenario a.

#### Variable selection

**Fig 3:**
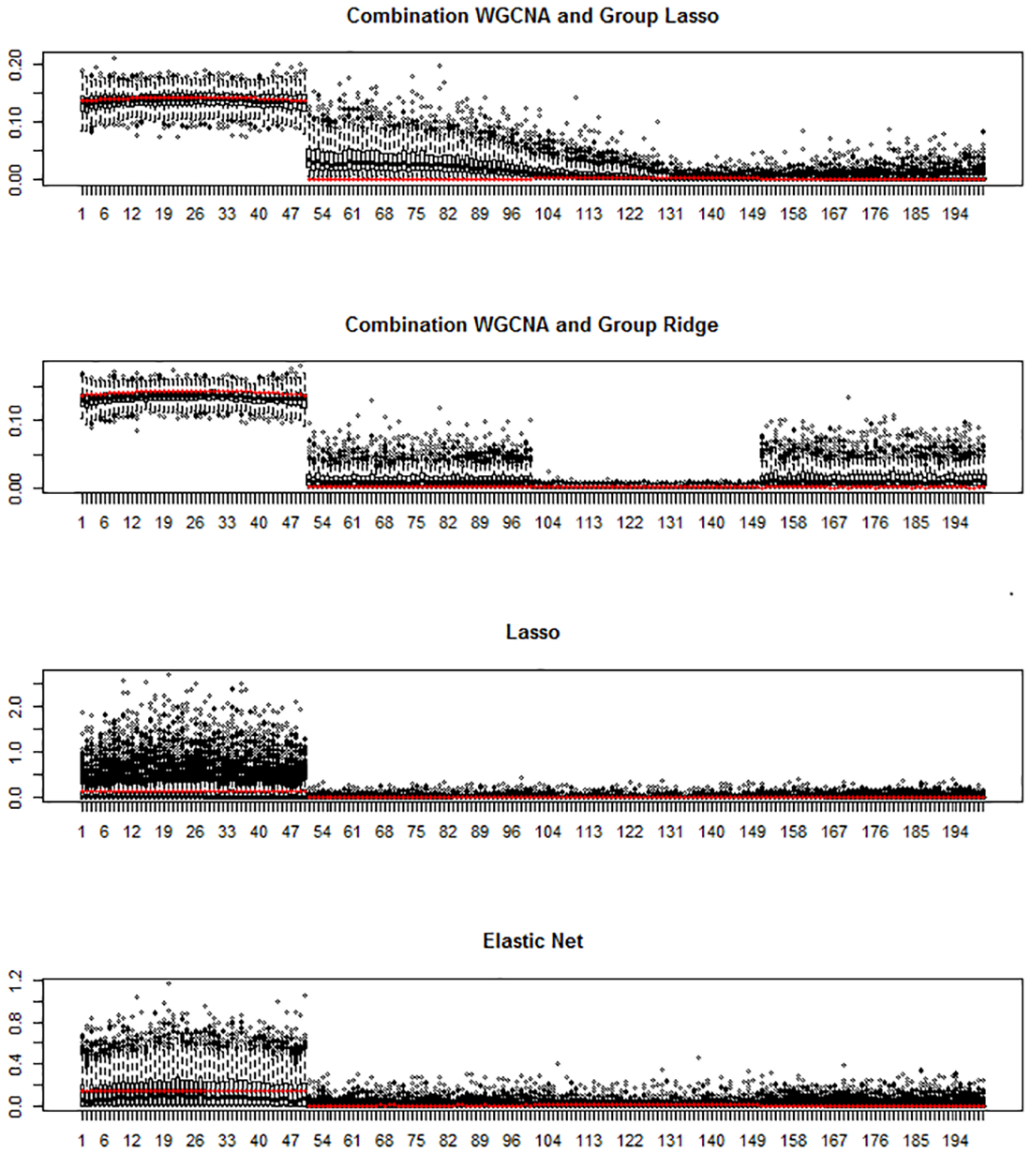
Simulation study: Variable selection results with WGCNA. Variable selection results for scenario a, *k* = 4, *p* = 200, and *n* = 100. Box-plots of the absolute values of the estimated parameters for the 200 variables over the 500 simulated datasets are plotted. The red points represent the absolute average true values over the 500 datasets.

**Fig 4:**
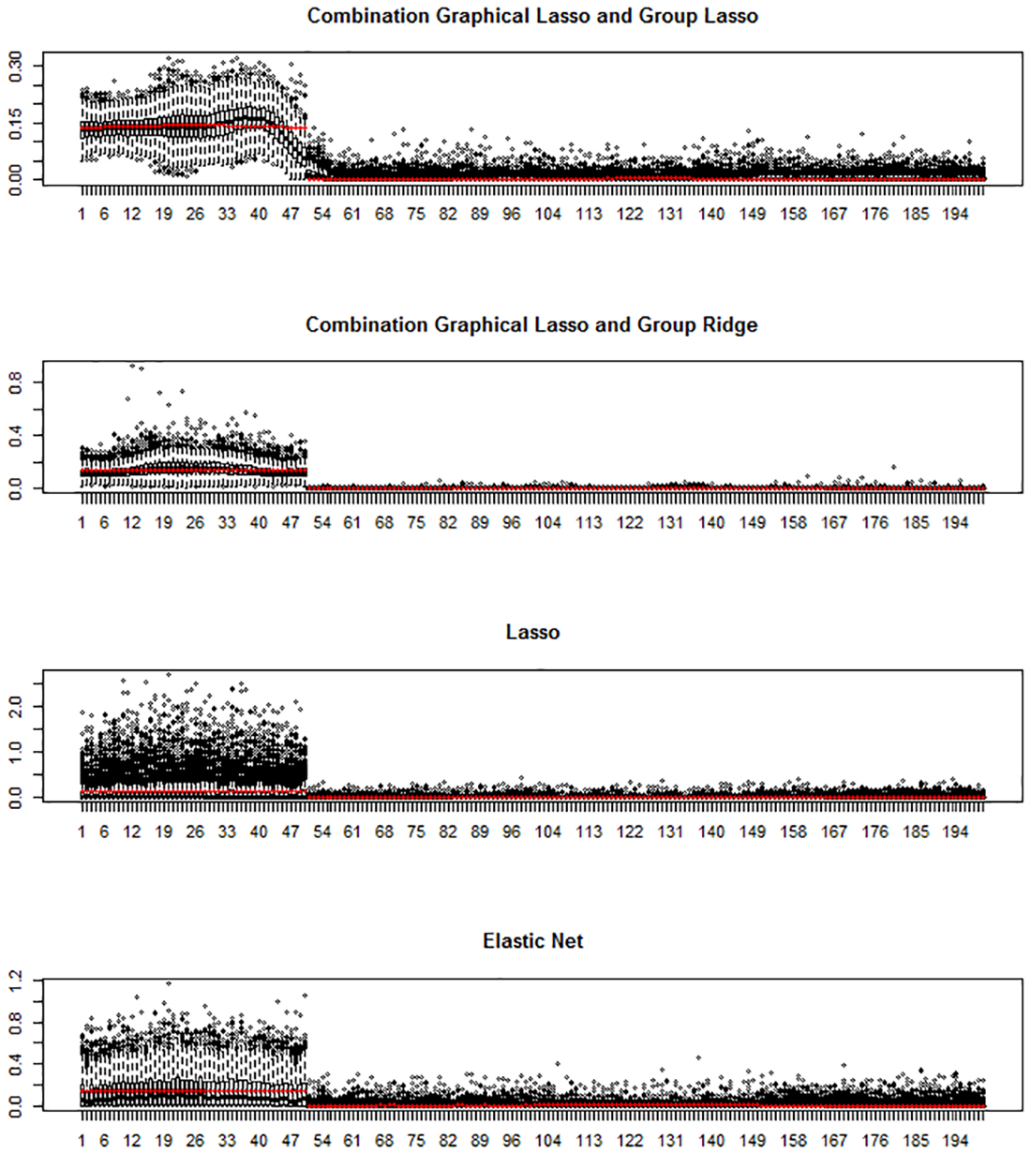
Simulation study: Variable selection results with graphical lasso. Variable selection results for scenario a, *k* = 4, *p* = 200, and *n* = 100. Box-plots of the absolute values of the estimated parameters for the 200 variables over the 500 datasets simulated are plotted. The red points represent the absolute average true values over the 500 datasets.

Finally, we investigated the variable selection properties of the best performing (in terms of predictive ability) three-step procedures. Figs 3 and 4 show for scenario a, *k* = 4, *p* = 200 and *n* = 100 the variable selection properties of adaptive group ridge and group lasso in combination with WGCNA and graphical lasso, respectively. In both cases, the performance of lasso and elastic net is also shown. For each method, each boxplot shows for each of the *p* variables of **X** the distribution of the average estimated regression coefficients over the 10 fold cross-validation folds for each of the *M* = 500 Monte Carlo trials. The true simulated regression coefficients are also shown (red dots). Complete results for all scenarios are presented in the S2 File, Fig A to Fig R.

These results show that our three step approaches perform well in terms of specific regression coefficient estimation and variable selection. The four investigated approaches given by the combination of WGCNA and graphical lasso with adaptive group ridge and group lasso clearly separate informative from non-informative variables. In contrast, lasso regression, especially in scenario a, shows a very poor performance. The mean estimated coefficients by lasso for all *p* variables are close to zero, while the variability is very high for the features with non-zero effects, reflecting that lasso randomly selects a few of the informative variables and assigns a very large effect to them. To a lesser extent, the same phenomenon is also observed for elastic net. Even if the mean estimate for informative variables is larger and variability is lower than for lasso, the overall performance of elastic net is inferior to our three-step methods based on including grouping information.

Fig 3 top panel shows that the combination of WGCNA and group lasso tends to overestimate the effect of the variables belonging to the second cluster of variables. This is due to the underestimation of the number of clusters by WGCNA and the joint penalization of group lasso. Interestingly, adaptive ridge is less affected by this issue. When using graphical lasso as network analysis method, the first informative group of variables is clearly separated from the rest, and the estimation is close to the theoretical one (Fig 4).

## Real data analysis

We analyzed data from the DILGOM study and from the breast cancer cell line pharmacogenomics dataset. In both cases, the aim is to obtain biological insights about the features which drive the prediction of BMI and treatment response.

In the DILGOM study we consider two omics datasets measured at baseline to predict the body mass index (BMI) after seven years of follow-up. Serum nuclear magnetic resonance (NMR) spectroscopy metabolites measures and gene expression profiles were considered. The analysed sample contained n = 258 individuals for which both types of omic measurements and the outcome of interest (log-transformed BMI) were available. In the breast cancer cell lines dataset, we were interested in using gene expression for predicting the response to the Erlotinib drug. Treatment response is measured using the GI50 index, a quantitative measure which measures the growth inhibitory power of the test agent. The analysed sample consisted of 45 breast cancer cell lines.

### DILGOM: Metabolites

The serum metabolomic data consists of quantitative information on 57 metabolic measure of various types, including lipids, lipoprotein subclasses, amino acids, cholesterol, glycolysis-related metabolites and fatty acids (see S3 File, Table A). Tables 5 and 6 show the main results for the prediction of BMI after 7 years of follow-up using serum NMR metabolites as predictors. Table 5 shows the performance of each method in terms of predictive ability measured through *Q*^2^. We observe that adaptive group ridge and group lasso provide the best results and that they perform slightly better than ridge, lasso and elastic net. Namely, for adaptive group ridge when using graphical lasso *Q*^2^ = 0.244 and for adaptive group ridge in combination with WGCNA *Q*^2^ = 0.233, while for ridge *Q*^2^ = 0.227 and for lasso *Q*^2^ = 0.222. Also, group lasso combined with WGCNA outperforms ridge and lasso (*Q*^2^ of 0.241). Variable selection based on hubs presents a notably lower predictive ability (best performance is reached with graphical lasso, *Q*^2^ = 0.176) than methods based on regularization, except for sparse group lasso, which is not competitive at all (*Q*^2^ < 0.002 in all cases). Table 6 shows the variable selection properties of the two top performing methods; the combination of WGCNA and group lasso and the combination of graphical lasso and adaptive group ridge. The top 12 variables selected by the combination of WGCNA and group lasso approach are shown in the left part of Table 6, jointly with their average regression coefficient, selection frequency over the 10 cross-validation folds used in the analysis, and their cluster membership. For each of these top 12 variables, average effect and selection frequencies over the 10 cross-validation folds are also shown for the combination of graphical lasso and adaptive group ridge, lasso, and elastic net. These top 12 variables represent two different families of metabolites. Namely, lipids and fatty acids (XSVLDLL, XLHDLL, SM, SHDLL, FAW6), and amino-acids and glycolysis-related metabolites (ALB, TYR, PHE, GLY, GLOL, GLC). This means that the three-step approach based on WGCNA and group lasso consistently points out these groups of metabolites as those driving the prediction of BMI. Accordingly, these two families of metabolites are well separated in the network analysis plus clustering steps (by both WGCNA and graphical lasso methods), consistently belonging to different clusters (see columns labeled ‘Cluster’ in Table 6).

**Table 5:** DILGOM metabolomics. Prediction accuracy of the models obtained for the different approaches on metabolites. In bold are the combinations of network analyses and prediction approaches which perform better than lasso, ridge, and elastic net.

	WGCNA	Graphical lasso	Ridge penalty
	*Q*^2^	*Q*^2^	*Q*^2^
Hubs + ridge	0.153	0.176	0.153
Group lasso	**0.241**	0.225	0.221
Sparse group lasso *α* = 0.5	0.013	0.010	0.015
Sparse group lasso *α* = 0.9	0.003	0.012	0.013
Sparse group lasso *α* = 0.1	0.013	0.007	0.016
Group ridge	**0.233**	**0.244**	0.225
Lasso	0.227	0.227	0.227
Ridge	0.222	0.222	0.222
Elastic net	0.208	0.208	0.208
Number of Clusters	4	7	4-6

**Table 6:** DILGOM metabolomics. Top 12 metabolites (in terms of average beta) selected by the combination of WGCNA and group lasso, their selection frequencies and cluster membership. For lasso, graphical lasso + ridge, and elastic net, the rank of the variables according to the absolute values of the average effect size is added.

	WGCNA + Group lasso			Graphical lasso + adaptive group ridge		
Variable	Average beta	Frequency	Cluster	Average beta	Rank	Cluster
GLOL	.064	10	1	.039	5	6
TYR	.060	10	1	.070	2	1
ALB	-.059	10	1	-.075	1	1
GLY	-.041	10	1	-.039	4	1
PHE	.038	10	1	.046	3	1
XSVLDLL	.038	10	2	.017	16	2
XLHDLL	-.038	10	3	-.034	7	5
HIS	-.036	10	1	-.030	8	1
SM	.034	10	2	.016	17	2
FAW6	.031	10	2	.003	31	3
GLC	.031	10	1	.037	6	1
SHDLL	.030	10	2	.030	9	5
	Lasso			Elastic Net		
	Average beta	Frequency	Rank	Average beta	Frequency	Rank
GLOL	.074	10	4	.063	10	3
TYR	.080	10	3	.068	10	2
ALB	-.086	10	2	-.069	10	1
GLY	-.037	10	6	-.035	10	7
PHE	.038	10	5	.042	10	5
XSVLDLL	.036	10	7	.038	10	6
XLHDLL	-.089	9	1	-.056	10	4
HIS	-.024	9	8	-.020	10	11
SM	.018	8	10	.011	8	17
FAW6	.017	7	12	.011	8	14
GLC	.018	10	11	.022	10	9
SHDLL	.003	3	20	.005	7	20

Interestingly, our three-step approach based on the combination of WGCNA and group lasso provides similar effect estimates for metabolites XSVLDLL, SM, FAW6 and SHDLL (.038, .034, .031, and.030, respectively), all of them belonging to the same cluster of lipids and fatty acids. The combination of graphical lasso and adaptive group ridge provides similar results in terms of effect size. On the contrary, lasso provides more extreme estimates due to within-group random variable selection, i.e. lasso selects at random oen feature over a set of highly correlated variables. Specifically, lasso assigns quite different effect estimates to the lipids and fatty acids group (XSVLDLL: .036, SM: .018, FAW6: .017, SHDLL: .003). The effect size of SHDLL is particularly counter-intuitive since high density lipids are well established risk factors for obesity [39]. Elastic net appears not to solve this issue and provides similar results than lasso.

### DILGOM: Transcriptomics

Due to the computational intensity of the graphical lasso approach, we considered two sets of gene expression probes for analysis. A set of 2980 probes which was only analysed by WGCNA to perform network analysis and a set of 732 filtered probes (probes with a variance higher than 1) were WGCNA and graphical lasso were used. The main results are presented in Tables 7 and 8. Table 7 presents the prediction ability results of the used methods. For the set of filtered probes (left part of Table 7), the best method with regard to predictive performance is the combination of WGCNA and group lasso (*Q*^2^ = 0.258). Adaptive group ridge appears to provide poor results (*Q*^2^ = 0.158 in combination with WGCNA and *Q*^2^ = 0.188 in combination with graphical lasso) in the transcriptomics context. In contrast to the observed results regarding the NMR metabolites, adaptive ridge is clearly outperformed by lasso (*Q*^2^ = 0.227) and elastic net (*Q*^2^ = 0.253), but still provide better results than the ridge regression (*Q*^2^ = 0.071). Also, we observe that for transcriptomics elastic net provides better results than lasso which was not the case for the metabolites. For the larger set of probes (right part of Table 7), the best prediction accuracy is achieved using the combination of WGCNA with group lasso *Q*^2^ = 0.418 while lasso and elastic net show similar predictive abilities with *Q*^2^ = 0.257 and *Q*^2^ = 0.265, respectively. Ridge presented better prediction accuracy with the large set of probes but its performance is still very low (Q2 = 0.131). In line with the simulation study, the benefits of our three-step proposal is larger when the number of probes increases.

**Table 7:** DILGOM transcriptomics. Prediction accuracy of the models obtained by combination of networks and prediction models as well as lasso, ridge, and elastic net for transcriptomics.

	Filtered set (p = 732)		Larger set (p = 2980)
	WGCNA	Graphical lasso	WGCNA
	*Q*^2^	*Q*^2^	*Q*^2^
Group lasso	0.258	0.215	0.418
Group ridge	0.158	0.188	
Lasso	0.227	0.227	0.257
Ridge	0.071	0.071	0.131
Elastic net	0.253	0.253	0.265
Number of clusters	16-17	32-36	40-45

**Table 8:** DILGOM transcriptomics. Number of variables selected during the cross-validation process, at least once, in all croos-validation folds and the proportion of variables selected all in the set of variables selected at least once.

	Filtered set (p = 732)			Larger set (p = 2980)		
	Always	At least once	Proportion	Always	At least once	Proportion
WGCNA and group lasso	137	687	0.199	48	252	0.190
Graphical lasso and group lasso	92	485	0.189			
Lasso	3	78	0.038	13	134	0.097
Elastic net	7	123	0.056	21	176	0.119

Table 8 presents the number of variable selected for the two group lasso approaches (based on WGCNA and graphical lasso), lasso, and elastic net. The left part of Table 8 shows the results for the filtered set of probes and the right part shows the results for the large set of probes. For the filtered set of probes, it appears that group lasso retains more variables than lasso and elastic net. WGCNA in combination with group lasso provided 687 variables which were selected at least once during the cross validation process, while the combination of graphical lasso and group lasso provided 485 variables. Lasso and elastic net identified only 78 and 123 variables, respectively. Moreover, the models obtained with group lasso are more stable than those obtained with the standard approaches, lasso and elastic net. Indeed, using WGCNA, 19.9% of the 687 variables are selected in all the 10 cross-validation folds and for graphical lasso 18.9% of the 485 variables are selected. In contrast, for lasso and elastic net only 3.8% and 5.6% of the variables are selected in all the cross-validation folds. For the larger set of probes, the number of variables always selected increased for lasso and elastic net with respectively 13 and 21 variables, this is not the case for the combination of WGCNA with group lasso with 48 variables always selected for the set of 2928 probes while 137 variables were always selected with the smaller set of probes. From the 48 variables obtained, only 5 were also included in the previous set of 137 variables.

To investigate the biological relevance of the selected variables in the prediction models obtained, a gene set enrichment analysis was performed using the Gene Set Enrichment Analysis software (GSEA) [40, 41] on the variables always selected by each approach during the cross-valiadation process. A gene set enrichment analysis consists of comparing the set of gene identified with a priori known group of genes that have been grouped together by their involvement in the same biological pathway. Table 9 presents the results of the enrichment analysis when using the large set of transcriptomics. None of the pathways obtained in the enrichment analysis by the different methods has been previously identified as related to BMI. The enrichment analysis based on the 137 and 92 genes obtained from the filtered set of probes was more insightful. Among the 137 genes selected by the combination WGCNA and group lasso, 33 were associated with cardiovascular disease (*p* = 0.019) and 6 of these 33 genes were associated with obesity (*p* = 0.044). Among the 92 genes obtained with the combination of graphical lasso and group lasso, 3 of them where included in the glucagon signaling pathway (*p* = 0.070) and 3 were in the insulin resistance pathway (*p* = 0.080). These results are not surprising since it is known that increased insulin and decreased glucagon secretion play a role in obesity [42]. Due to the small number of variables of lasso and elastic net, 7 and 3 predictors respectively, the enrichment analysis did not provide associated pathways.

**Table 9:** DILGOM transcriptomics. Top significant pathways identified by enrichment analysis using the GSEA software for all predictions model using the variables always selected during the cross-validation process of the breast cancer cell lines study on the transcriptomics data. For each method, the number of variables common to the pathway and the set of variables selected at least 5 times and the false discovery rate (FDR) of the enrichment test are presented.

method	Pathway	Number variables	FDR
WGCNA and group lasso	Genes transcriptionally modulated in the blood of multiple sclerosis patients in response to subcutaneous treatment with recombinant IFNB1	10	9.68 e-15
	Genes up-regulated in CD34+ hematopoetic cells by expression of NUP98-HOXA9 fusion off a retroviral vector at 3 days after transduction	10	3.86 e-12
	Genes representing interferon-induced antiviral module in sputum during asthma exacerbations	8	1.27 e-11
Lasso	Genes exclusively down-regulated in B lymphocytes from WM (Waldenstroem’s macroblobulinemia) patients but with a similiar expression pattern in the normal cells and in the cells from CLL (chronic lymphocytic leukemia) patients.	2	5.62 e-3
	Genes down-regulated in erythroid progenitor cells from fetal livers of E13.5 embryos with KLF1 knockout compared to those from the wild type embryos	6	5.62 e-3
Elastic net	Genes down-regulated in CD4+ T lymphocytes transduced with FOXP3.	3	1.55 e-3
	Genes up-regulated in MCF7 cells (breast cancer) after stimulation with NRG1	4	1.55 e-3
	Genes down-regulated in normal hematopoietic progenitors by RUNX1-RUNX1T1 fusion	4	1.55 e-3

### Breast cancer cell lines

The main results of the prediction of the treatment response of breast cancer cell lines to Erlotinib are presented in Table 10. The best prediction performance is again the combination of WGCNA and group lasso with *Q*^2^ = 0.654. Ridge with *Q*^2^ = 0.610 performs better than lasso and elastic net with *Q*^2^ = 0.571 and *Q*^2^ = 0.564, respectively. For this dataset the combination of WGCNA and group lasso is less stable and is not always able to pick the same variables during the cross-validation process, while lasso and elastic net are able to always pick 2 probes. With regards to variables selected at least 5 times by WGCNA + group lasso, lasso and elastic net, all 3 methods have a similar number of selected variables with respectively 22, 18 and 25. The intersection between 3 identified sets of variables is empty. The enrichment analysis identified genes related to breast cancer for the WGCNA + group lasso and elastic net approaches as presented Table 11. This was not the case for lasso.

**Table 10:** Breast cancer analysis. Prediction accuracy and numbers of variable selected at least 5 times and always selected in the 10-fold cross-validation process of the different approaches on the whole set of probes for the Breast cancer cell lines.

	*Q*^2^	Number of Variables	
		At least 5 times	always
WGCNA and group lasso	0.654	22	0
Lasso	0.571	18	2
Ridge	0.610	5376	5376
Elastic net	0.564	25	2
Total number of variables		5376	5376

**Table 11:** Breast cancer analysis. Top significant pathways identified by enrichment analysis using the GSEA software for all predictions model using variables selected at least 5 times during the cross-validation process on the transcriptomics data of the breast cancer cell lines study. For each method, the number of variables common to the pathway and the set of variables selected at least 5 times and the false discovery rate (FDR) of the enrichment test are presented.

method	Pathway	Number variables	FDR
WGCNA and group lasso	Candidate genes in genomic amplification regions in hepatocellular carcinoma (HCC) samples	6	5.61 e-10
	Genes within amplicon 17q11-q21 identified in a copy number alterations study of 191 breast tumor samples.	6	6.49 e-8
	Genes up-regulated in DLBCL (diffuse large B-cell lymphoma) cell lines sensitive to stimulation of CD40 relative to the resistant ones	5	4.62 e-5
Lasso	Genes up-regulated in confluent IMR90 cells (fibroblast) after knockdown of RB1 by RNAi	7	5.621 e-6
	Genes up-regulated in the neural crest stem cells (NCS), defined as p75+/HNK1+	5	5.92 e-6
	Genes down-regulated in BEC (blood endothelial cells) compared to LEC (lymphatic endothelial cells)	5	6.66 e-6
Elastic net	Genes down-regulated in TMX2-28 cells (breast cancer) which do not express ESR1 compared to the parental MCF7 cells which do	11	5.41 e-10
	Genes up-regulated in confluent IMR90 cells (fibroblast) after knockdown of RB1 by RNAi.	9	2.52 e-8
	Genes positively correlated with recurrence free survival in patients with hepatitis B-related (HBV) hepatocellular carcinoma (HCC)	5	4.57 e-6

## Discussion

In this paper, we presented a new strategy to obtain accurate, stable and interpretable prediction models. The key components of our proposed approach are to capture the correlation structure of the features within an omic dataset, to derive clustering information, and to include it in a group penalization model. Our approach seems to provide interpretable models by capturing underlying biological mechanisms impacting the phenotype of interest.

Our applications showed that the proposed three step approach can outperform the standard regularized regression approaches in terms of prediction ability, stability and biological interpretation in high-dimensional settings or when groups of strongly correlated features are present in the data. Our analyses highlighted the weakness of methods such as lasso and elastic net in terms of stable variable selection in highly correlated datasets. Indeed, for the metabolites, our WGCNA and group lasso combination selected a group of highly correlated metabolites (cluster 2 including XSVLDLL, SM, FAW6, and SHDLL) while lasso selected XSVLDLL all the times in the cross-validation process but SHDLL only 3 out of 10 times. In addition it appeared that for the large transcriptomics dataset the prediction accuracy is also larger for our proposed methods than for the standard regularization methods. The analysis of the breast cancer cell lines study showed some limitations in terms of stability for our network-based approach when the number of samples is relatively small. Probably the networks obtained during the cross-validation steps are less stable for a small number of samples leading to a less stable clustering and prediction model. Further with regard to transcriptomics, the obtained groups of gene expression features identified by our strategies were enriched for known pathways linked to BMI (DILGOM) and breast cancer (breast cancer cell lines). This was only the case when using the filtered transcriptomics dataset. This was not always the case for lasso, ridge, and elastic net. For the unfiltered transcriptomic datasets, the gene sets were not enriched for pathways related to the outcome. Here more research is needed. These results suggest that our proposed approaches can indeed improve the understanding of prediction models while keeping a good prediction accuracy.

The performance of our approaches compared to the standard approaches was in line with the results obtained from the simulation study. Indeed the combination of WGCNA or graphical lasso with group lasso appeared to provide the most stable results, hence probably better interpretable. The prediction accuracy of these approaches was also good and for large omics datasets even better than the prediction accuracy of the standard approaches. Further our simulations showed that several group penalization models (sparse group lasso and adaptive group ridge) are quite sensitive to the used grouping structure. In contrast the group lasso approach proved to be quite robust with respect to the network approach used. Also, we have explored the idea of reducing the omic dataset dimensionality by choosing ‘important’ features by group based on network topology (such as our ‘hubs’ selection). This attractive approach to reduce the prediction complexity only performed well when using WGCNA for predictors which are highly associated to the phenotype of interest. Its performance was very sensitive to the used network method and bad in low-signal situations. Overall the combination of graphical lasso for network construction and group lasso was the best performing method in our simulation study. However, this approach computationally challenging for a large number of features and, therefore, cannot deal with large omics datasets. Moreover in the real data analysis better results were obtained when WGCNA was combined with group lasso. Therefore, for large datasets we recommend the combination of WGCNA and group lasso, while for smaller datasets both network approaches can be applied.

The presented work can be extended in various ways. So far, all our analyses focused on prediction of a continuous outcome, but all the obtained results apply, in principle, to other types of response variables, such as binary outcomes (classification problems) and to time-to-event data. Also, prior knowledge on biological grouping could be included in our three-step approaches if available, even if it is only partial. Our simulation study showed good results if the correct underlying clustering is known. Given that such biological knowledge is only partially known in many omic applications, we have proposed to use network analysis to infer the correlation structure. Including external prior biological in the first step of network construction may lead to an improvement of the clustering obtained and, therefore, of the proposed methods. Another possible extension is to build prediction models with two or more sets of omic predictors. It is known [1] that using a common penalization (such as lasso or ridge) to the extended dataset containing both omic sets to be combined can lead to worse predictive ability than using only one of these omic sets. Therefore, applying our three-step approach to the stacked dataset of different omic predictors may outperform current methods. Alternatively, more advanced network techniques as multi-layer networks [43], based on obtaining the correlation structure between and within the omic sets may be improve prediction models. These extensions are currently under investigation.

To conclude, we presented a set of methods which provides accurate and stable predictions possibly leading to better interpretation, as is shown in the real data application. In the DILGOM study, a much more stable set of metabolomic predictors for BMI was obtained compared to standard approaches. Moreover, better predictions were obtained with our approach when using a large set of gene expression probes to predict BMI. Regarding the prediction of breast cancer, identified gene modules with our approach appeared to be interpretable since enrichment analyses showed that selected features could be linked with breast cancer tumors. This was not the case when using the standard approaches.

## Ethics statement

The DILGOM data and the breast cancer cell data lines were fully anonymized before the authors could access them.

## Supporting information

S1 File Prediction accuracy results for *n* = 100 individuals. Results in terms of predictive ability for simulated datasets of 100 individuals for 200 variables and 1000 variables.
(PDF)

S2 File Complete variable selection results. Boxplots of estimated effects of each of the *p* variables of **X** and the distribution of the average estimated regression coefficients for all the combinations of network an group penalization prediction methods.
(PDF)

S3 File List of metabolites used in the DILGOM analysis. Detailed description and acronyms of the 57 metabolites studied in the DILGOM study.
(PDF)
