# Supplementary material for: Improving stability of prediction models based on correlated omics data by using network approaches
Source: PLoS One. 2018 Feb 20;13(2):e0192853. doi: 10.1371/journal.pone.0192853 (PMC5819809; doi:10.1371/journal.pone.0192853)
Supplement: S1 File — Results in terms of predictive ability for simulated datasets of 100 individuals for 200 variables and 1000 variables. (PDF) [file pone.0192853.s001.pdf]

S1 File : Prediction accuracy results for 100 individuals.

Renaud TISSIER, Jeanine HOUWING-DUISTERMAAT, Mar RODRÍGUEZ-GIRONDO

A simulation study has been set up to evaluate the performance of our proposed three-step methods in various settings and to compare them with the standard approaches lasso, ridge and elastic net. This supplementary Information shows the results in terms of predictive ability for simulated datasets of 100 individuals.

Table A: Results obtained in terms of Q_2 for the 3 scenarios, 200 variables, 4 and 8 modules, and 100 individuals. Into brackets are the standard errors

		4 modules			8 modules		
		Scenario a	Scenario b	Scenario c	Scenario a	Scenario b	Scenario c
A Priori	Sparse group $\text{lasso}_{0.5}$	.793(.004)	.588(.049)	.682(0.018)	.753(.012)	.729(.021)	.714(0.028)
	Sparse group $\text{lasso}_{0.9}$	.793(.004)	.565(.049)	.653(.019)	.751(.012)	.716(.022)	.689(0.033)
	Sparse group $\text{lasso}_{0.1}$	.793(.004)	.597(.049)	.687(.018)	.754(.012)	.733(.018)	.720(0.021)
	Group Lasso	.881(.004)	.602(.058)	.808(.014)	.849(.010)	.817(.022)	.836(0.017)
	Group Ridge	.955(.014)	.541(.059)	.787(.028)	.930(.011)	.834(.036)	.918(0.016)
WGCNA	Hubs	.818(.024)	.141(.069)	.581(.103)	.830(.030)	.583(.105)	.554(.12)
	Sparse group $\text{lasso}_{0.5}$	.751(.104)	.155(.121)	.647(.118)	.382(.226)	.305(.206)	.350(.210)
	Sparse group $\text{lasso}_{0.9}$	.752(.105)	.135(.229)	.607(.114)	.380(.224)	.284(.213)	.339(.203)
	Sparse group $\text{lasso}_{0.1}$	.736(.100)	.165(.120)	.651(.116)	.372(.225)	.318(.197)	.343(.203)
	Group Lasso	.923(.015)	.588(.071)	.921(.014)	.872(.028)	.816(.038)	.905(.014)
	Group Ridge	.877(.020)	.462(.063)	.788(.027)	.809(.040)	.768(.040)	.739(.059)
Graph Lasso	Hubs	.914(.017)	.520(.074)	.886(.035)	.860(.027)	.768(.020)	.887(.022)
	Sparse group $\text{lasso}_{0.5}$	.632(.149)	.072(.066)	.474(.179)	.298(.210)	.206(.141)	.290(.198)
	Sparse group $\text{lasso}_{0.9}$	.633(.146)	.068(.064)	.449(.172)	.311(.223)	.182(.143)	.284(.186)
	Sparse group $\text{lasso}_{0.1}$	.638(.145)	.072(.068)	.481(.181)	.297(.221)	.220(.141)	.288(.191)
	Group Lasso	.941(.011)	.633(.061)	.930(.011)	.894(.022)	.825(.034)	.918(.019)
	Group Ridge	.952(.014)	.591(.057)	.784(.026)	.894(.051)	.822(.042)	.796(.072)
Ridge Penalty	Hubs	.453(.145)	.117(.032)	.447(.120)	.121(.201)	.697(.081)	.309(.174)
	Sparse group $\text{lasso}_{0.5}$	.779(.041)	.433(.023)	.726(.015)	.171(.180)	.314(.164)	.300(.230)
	Sparse group $\text{lasso}_{0.9}$	.792(.008)	.474(.035)	.669(.020)	.166(.177)	.249(.187)	.249(.234)
	Sparse group $\text{lasso}_{0.1}$	.742(.068)	.410(.020)	.731(.013)	.166(.181)	.352(.165)	.294(.235)
	Group Lasso	.903(.018)	.512(.042)	.912(.012)	.864(.030)	.798(.044)	.897(.016)
	Group Ridge	.809(.022)	.152(.027)	.785(.026)	.758(.042)	.783(.049)	.758(.042)
Common approach	Lasso	.919(.016)	.551(.072)	.879(.017)	.865(.024)	.817(0.046)	.896(0.013)
	Ridge	.807(.022)	.156(.028)	.784(.026)	.673(.033)	.555(0.041)	.707(0.024)
	Elastic Net	.961(.04)	.743(.26)	.799(.20)	.879(.02)	.810(.04)	.895(.02)

Table B: Results obtained in terms of Q_2 for the 3 scenarios, 1000 variables, 4 and 8 modules, and 100 individuals. Into brackets are the standard errors

		4 modules			8 modules		
		Scenario a	Scenario b	Scenario c	Scenario a	Scenario b	Scenario c
A Priori	Sparse group lasso$_{0.5}$	.807(.001)	.680(.018)	.701(.034)	.781(.013)	.717(.026)	.758(0.018)
	Sparse group lasso$_{0.9}$	.806(.001)	.629(.021)	.638(.046)	.777(.014)	.675(.031)	.713(.033)
	Sparse group lasso$_{0.1}$	.807(.001)	.688(.018)	.718(.030)	.781(.012)	.725(.025)	.767(.016)
	Group Lasso	.896(.001)	.807(.014)	.793(.047)	.879(.008)	.840(.016)	.855(.011)
	Group Ridge	.978(.011)	.776(.027)	.614(.098)	.970(.011)	.930(.033)	.880(.027)
WGCNA	Hubs	.870(.017)	.581(.103)	.457(.324)	.438(.319)	.126(.108)	.107(.107)
	Sparse group lasso$_{0.5}$	.777(.100)	.683(.088)	.647(.118)	.439(.244)	.400(.225)	.357(.235)
	Sparse group lasso$_{0.9}$	.776(.102)	.622(.079)	.607(.114)	.443(.252)	.378(.215)	.325(.235)
	Sparse group lasso$_{0.1}$	.760(.098)	.686(.091)	.651(.116)	.408(.227)	.387(.216)	.368(.221)
	Group Lasso	.960(.004)	.915(.016)	.921(.014)	.883(.036)	.887(.018)	.883(.026)
	Group Ridge	.900(.014)	.779(.028)	.788(.027)	.707(.067)	.712(.046)	.803(.044)
Graph Lasso	Hubs	.618(.028)	.647(.035)	.886(.035)	.519(.044)	.544(.042)	.479(.036)
	Sparse group lasso$_{0.5}$	.799(.026)	.602(.090)	.474(.179)	.507(.216)	.367(.208)	.357(.198)
	Sparse group lasso$_{0.9}$	.799(.025)	.566(.084)	.449(.172)	.507(.216)	.358(.199)	.322(.198)
	Sparse group lasso$_{0.1}$	.801(.023)	.614(.088)	.481(.181)	.511(.223)	.373(.211)	.370(.190)
	Group Lasso	.971(.004)	.903(.012)	.930(.011)	.955(.009)	.916(.016)	.909(.019)
	Group Ridge	.975(.012)	.777(.027)	.784(.026)	.869(.099)	.760(.081)	.871(.034)
Ridge Penalty	Hubs	.010(.012)	.169(.053)	.093(.034)	.009(.012)	.112(.029)	.189(.043)
	Sparse group lasso$_{0.5}$	.098(.145)	.366(.323)	.027(.041)	.091(.112)	.317(.306)	.190(.194)
	Sparse group lasso$_{0.9}$	.122(.158)	.319(.297)	.020(.042)	.234(.253)	.279(.274)	.154(.207)
	Sparse group lasso$_{0.1}$	.082(.130)	.379(.328)	.031(.043)	.068(.090)	.314(.309)	.225(.184)
	Group Lasso	.970(.006)	.860(.016)	.702(.034)	.946(.014)	.901(.019)	.895(.025)
	Group Ridge	.944(.014)	.812(.026)	.484(.115)	.923(.015)	.898(.018)	.805(.033)
Common approach	Lasso	.949(.005)	.737(.032)	.701(.098)	.925(.014)	.816(.033)	.845(.035)
	Ridge	.860(.012)	.813(.018)	.384(.072)	.760(.024)	.744(.025)	.655(.031)
	Elastic Net	.951(.005)	.714(.043)	.735(.030)	.926(.013)	.830(.037)	.817(.033)
