# Supplementary material for: Improving stability of prediction models based on correlated omics data by using network approaches
Source: PLoS One. 2018 Feb 20;13(2):e0192853. doi: 10.1371/journal.pone.0192853 (PMC5819809; doi:10.1371/journal.pone.0192853)
Supplement: S2 File — Boxplots of estimated effects of each of the p variables of X and the distribution of the average estimated regression coefficients for all the combinations of network an group penalization prediction methods. (PDF) [file pone.0192853.s002.pdf]

S2 File : Complete variable selection results.

Renaud TISSIER, Jeanine HOUWING-DUISTERMAAT, Mar RODRÍGUEZ-GIRONDO

Contents

1 Scenario a **2**

1.1 4 modules 2

1.2 8 modules 6

2 Scenario b **10**

2.1 4 modules 10

2.2 8 modules 14

3 Scenario c **18**

3.1 4 modules 18

3.2 8 modules 22

A simulation study has been set up to evaluate the performance of our proposed three-step methods in various settings and to compare them with the standard approaches lasso, ridge and elastic net. Figures contained in this files show, for all the combinations of network an group penalization prediction methods, the boxplots of estimated effects of each of the p variables of $\mathbf{X}$ and the distribution of the average estimated regression coefficients over the 10 fold cross-validation folds for each of the M = 500 replicates.

1 Scenario a

1.1 4 modules

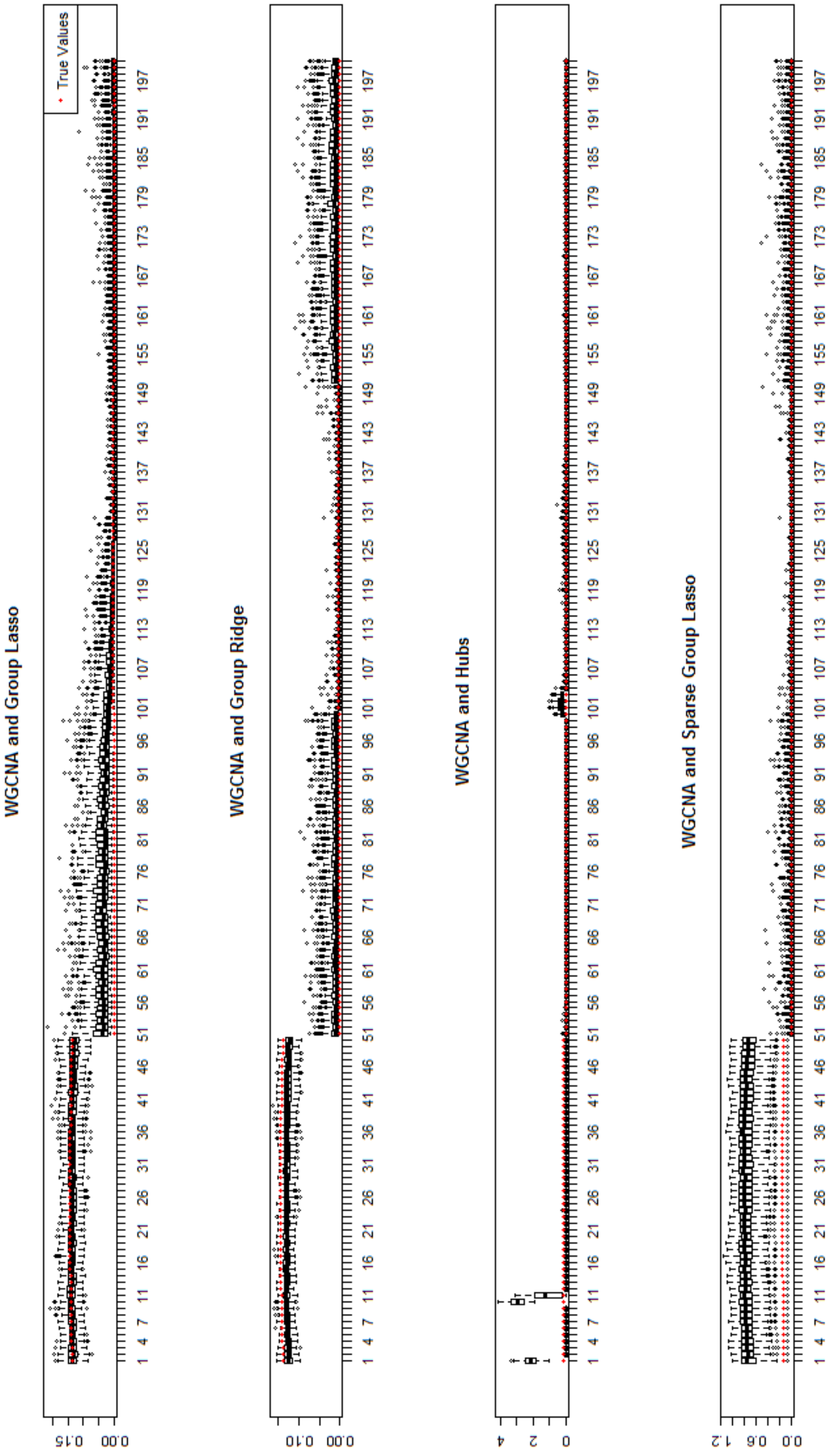

Figure A: Variable selection for scenario a, 8 modules, 200 variables and 50 samples. Box-plots of the absolute values of the estimated parameters for the 200 variables over the 500 datasets simulated are plotted. The red points represent the absolute average true values over the 500 datasets.

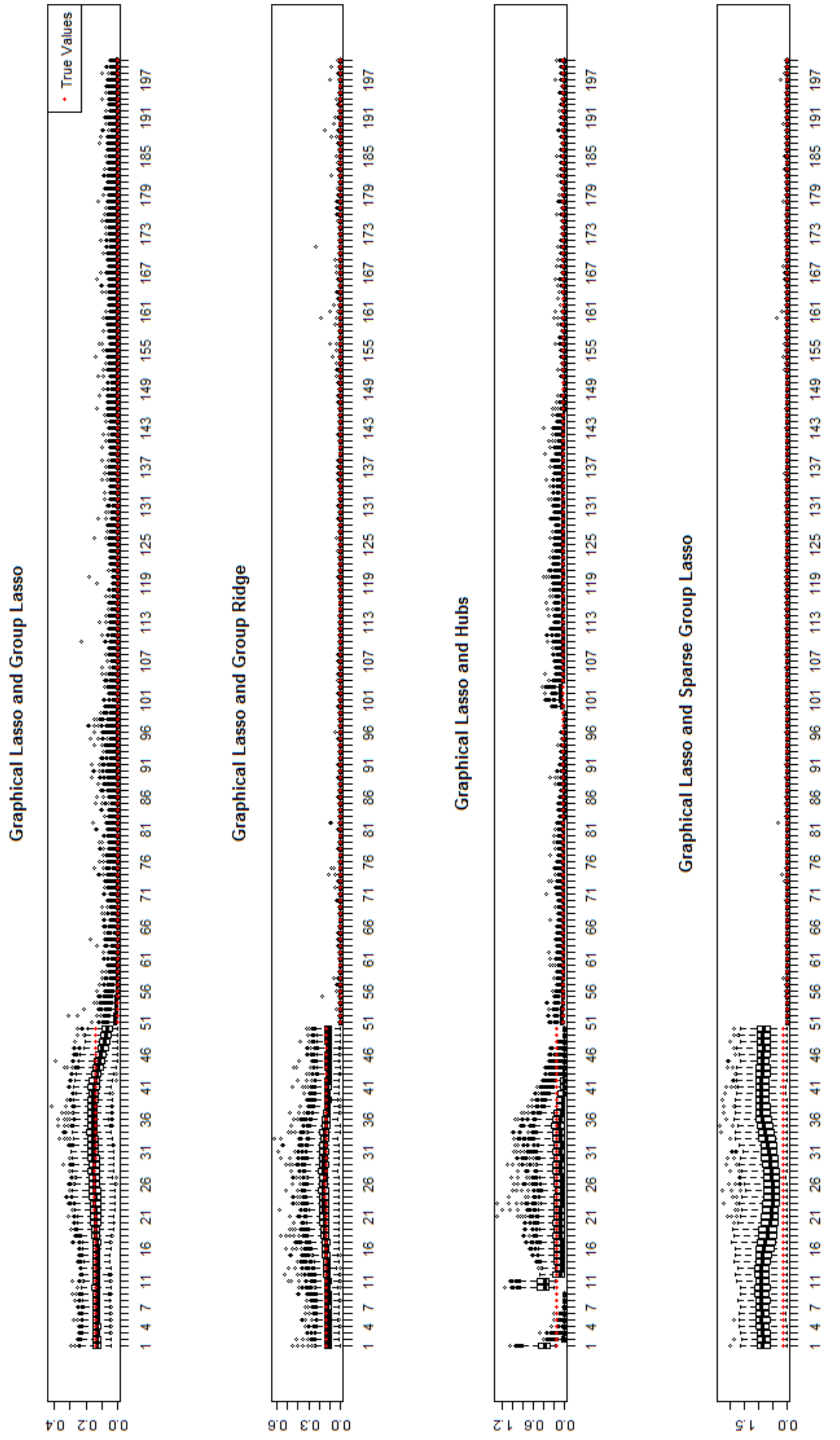

Figure B: Variable selection for scenario a, 8 modules, 200 variables and 50 samples. Box-plots of the absolute values of the estimated parameters for the 200 variables over the 500 datasets simulated are plotted. The red points represent the absolute average true values over the 500 datasets.

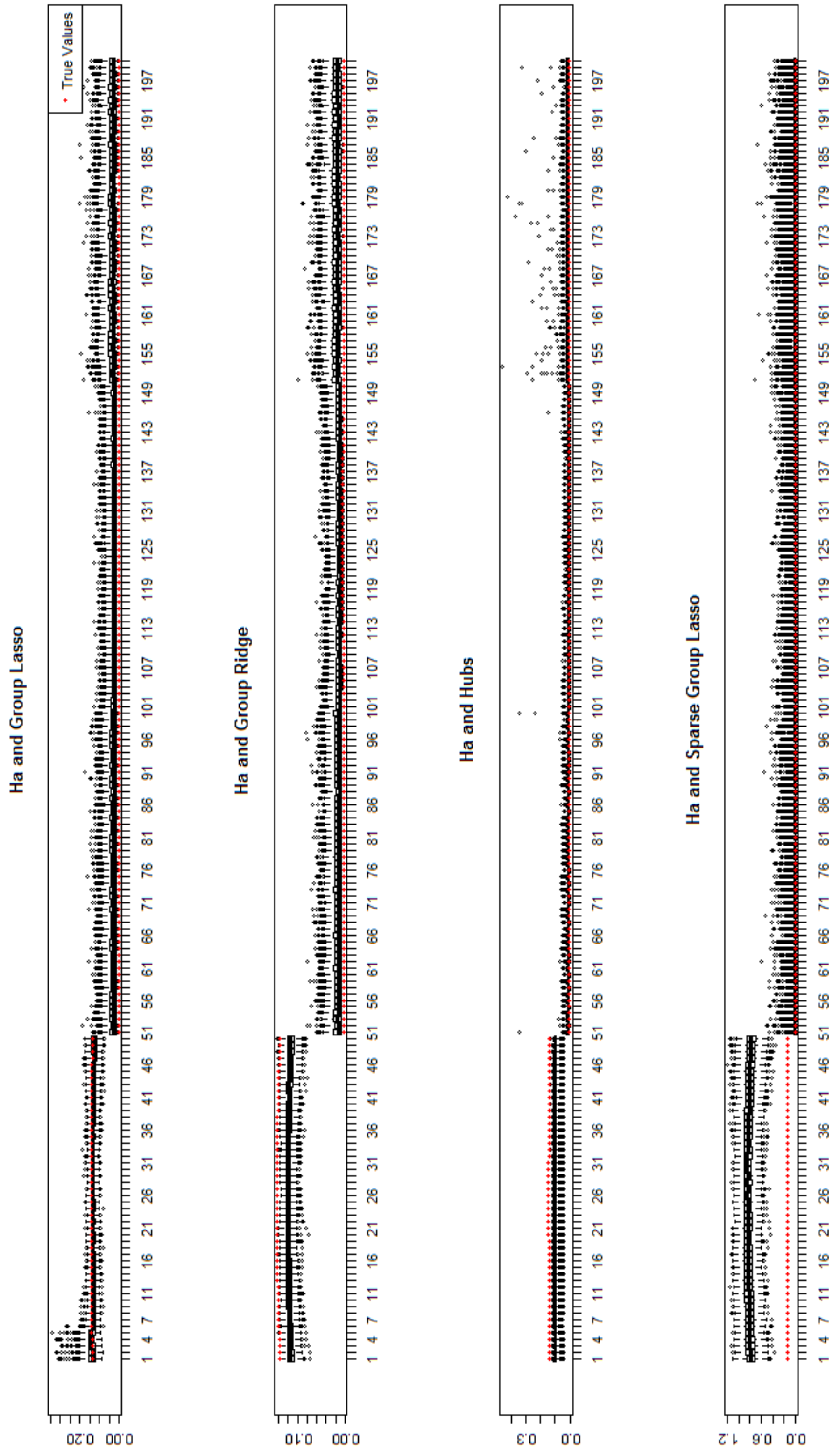

Figure C: Variable selection for scenario a, 8 modules, 200 variables and 50 samples. Box-plots of the absolute values of the estimated parameters for the 200 variables over the 500 datasets simulated are plotted. The red points represent the absolute average true values over the 500 datasets.

1.2 8 modules

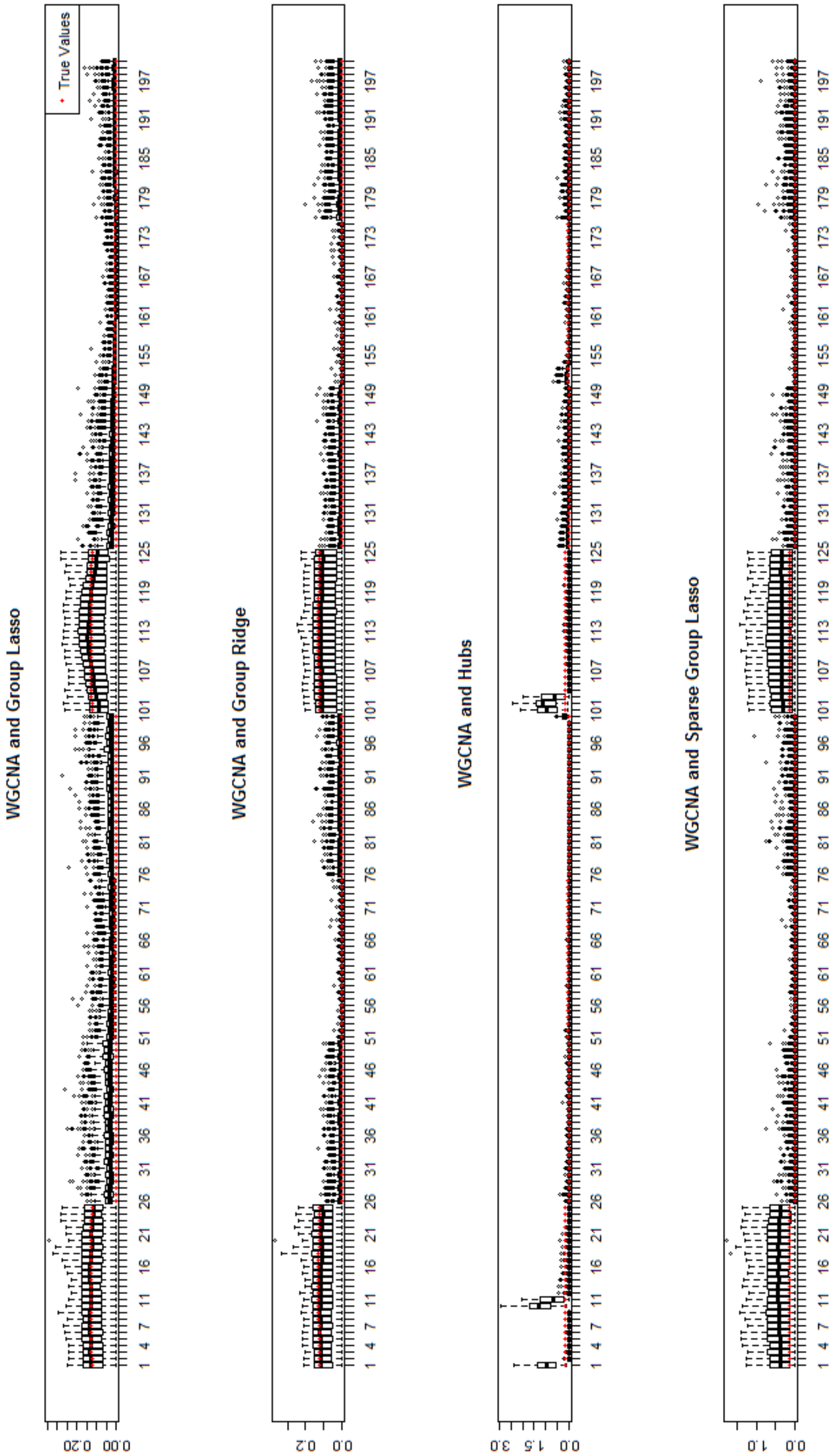

Figure D: Variable selection for scenario a, 8 modules, 200 variables and 50 samples. Box-plots of the absolute values of the estimated parameters for the 200 variables over the 500 datasets simulated are plotted. The red points represent the absolute average true values over the 500 datasets.

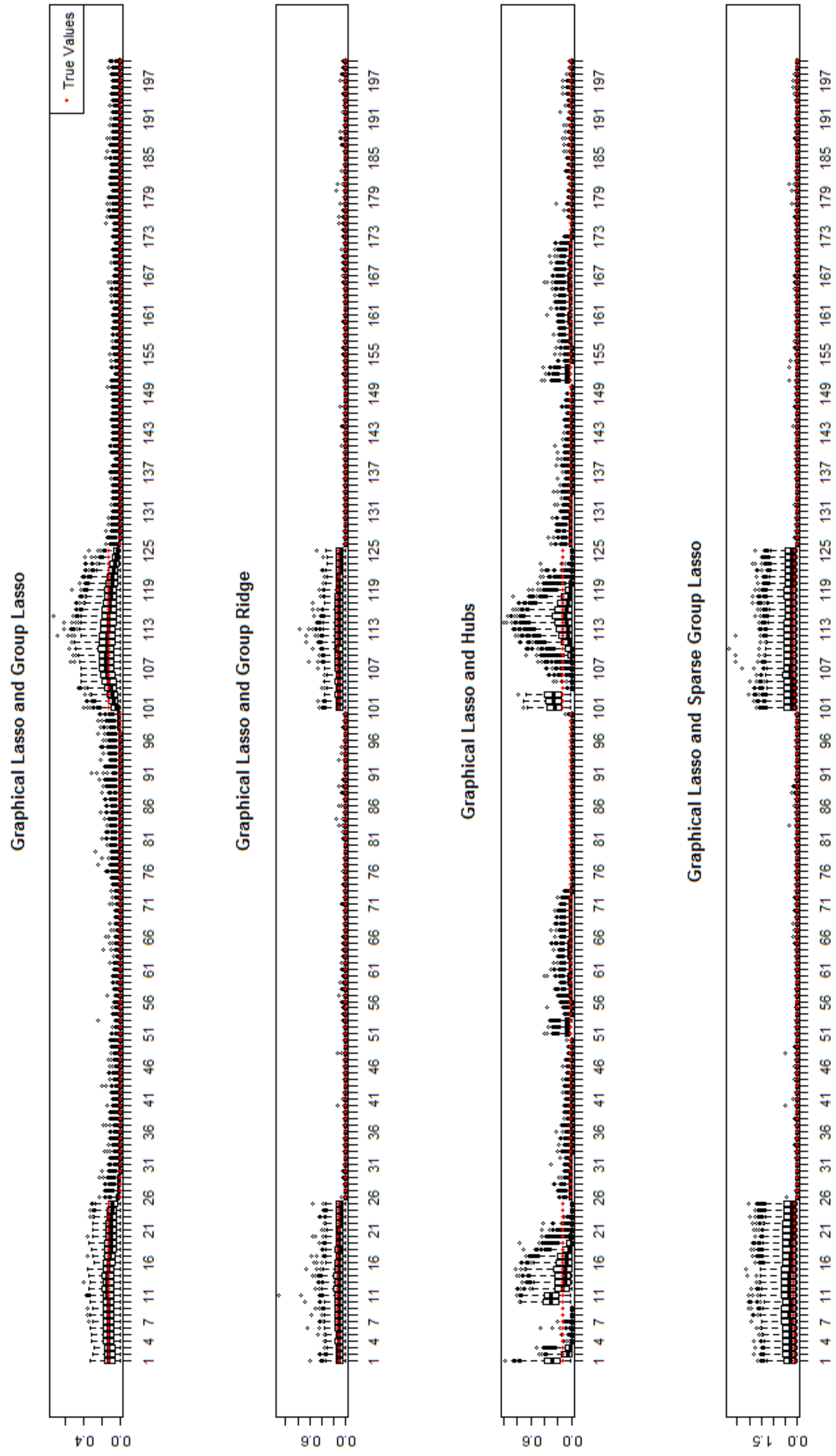

Figure E: Variable selection for scenario a, 8 modules, 200 variables and 50 samples. Box-plots of the absolute values of the estimated parameters for the 200 variables over the 500 datasets simulated are plotted. The red points represent the absolute average true values over the 500 datasets.

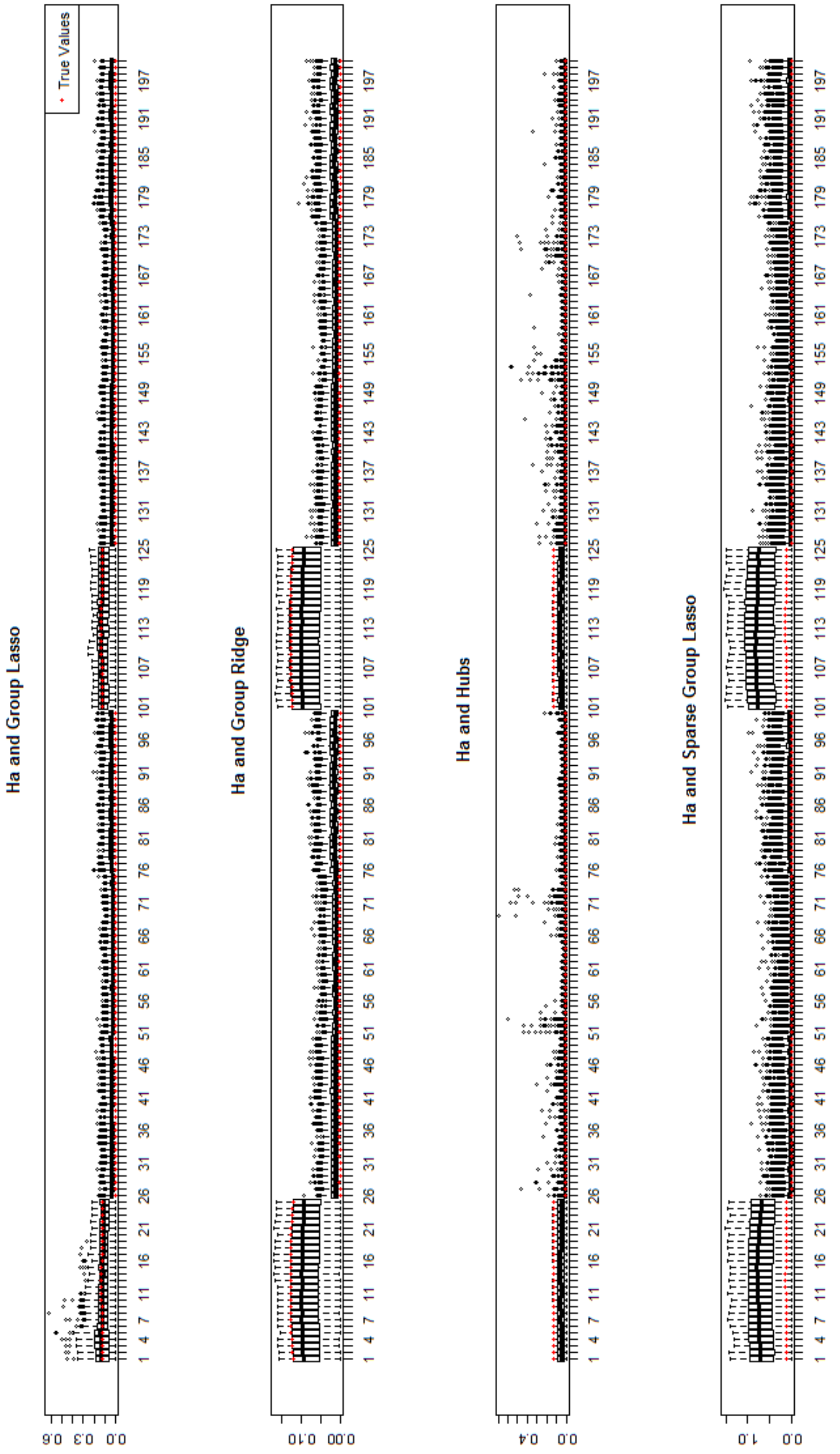

Figure F: Variable selection for scenario a, 8 modules, 200 variables and 50 samples. Box-plots of the absolute values of the estimated parameters for the 200 variables over the 500 datasets simulated are plotted. The red points represent the absolute average true values over the 500 datasets.

2 Scenario b

2.1 4 modules

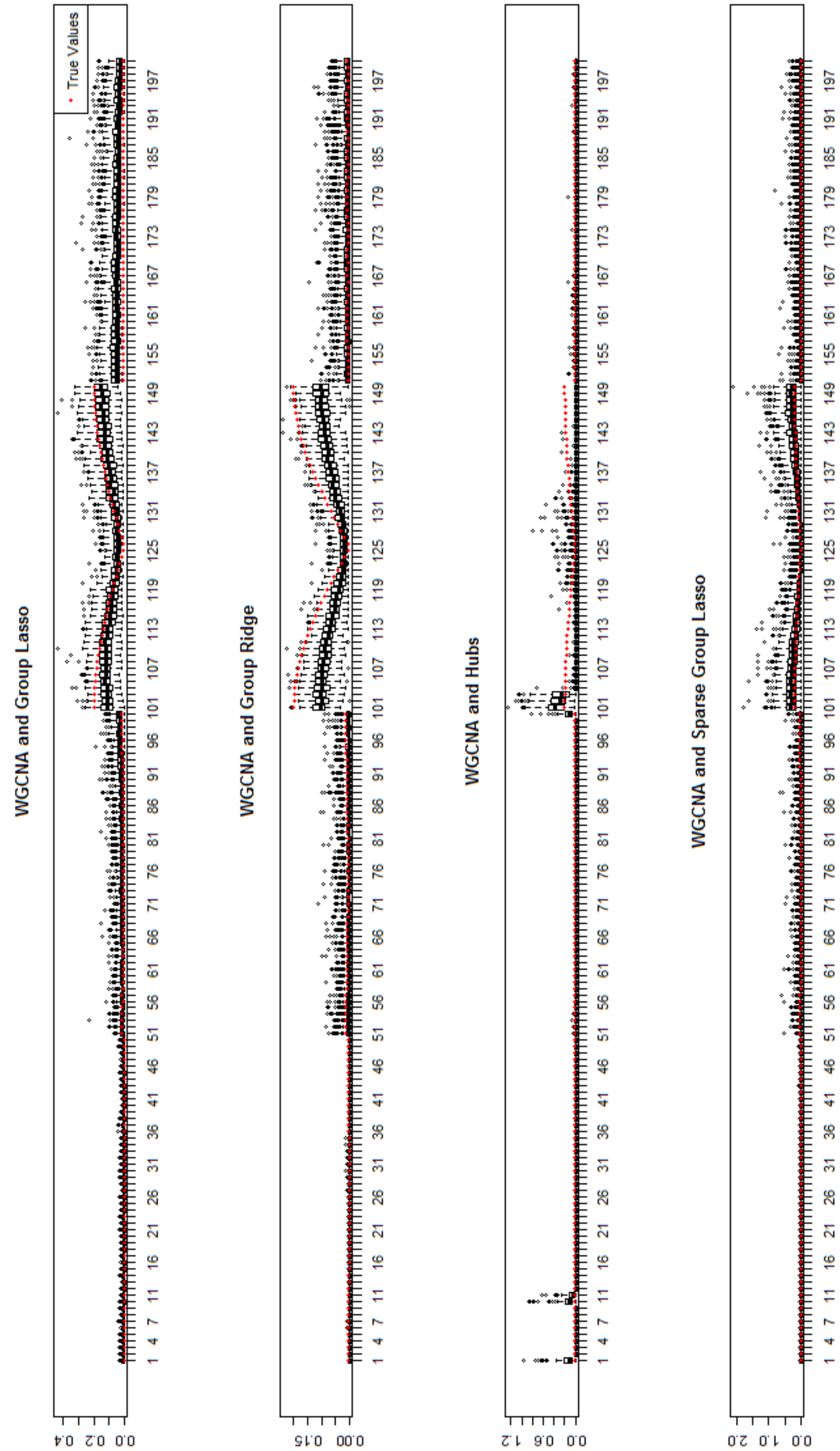

Figure G: Variable selection for scenario b, 4 modules, 200 variables and 50 samples. Box-plots of the absolute values of the estimated parameters for the 200 variables over the 500 datasets simulated are plotted. The red points represent the absolute average true values over the 500 datasets.

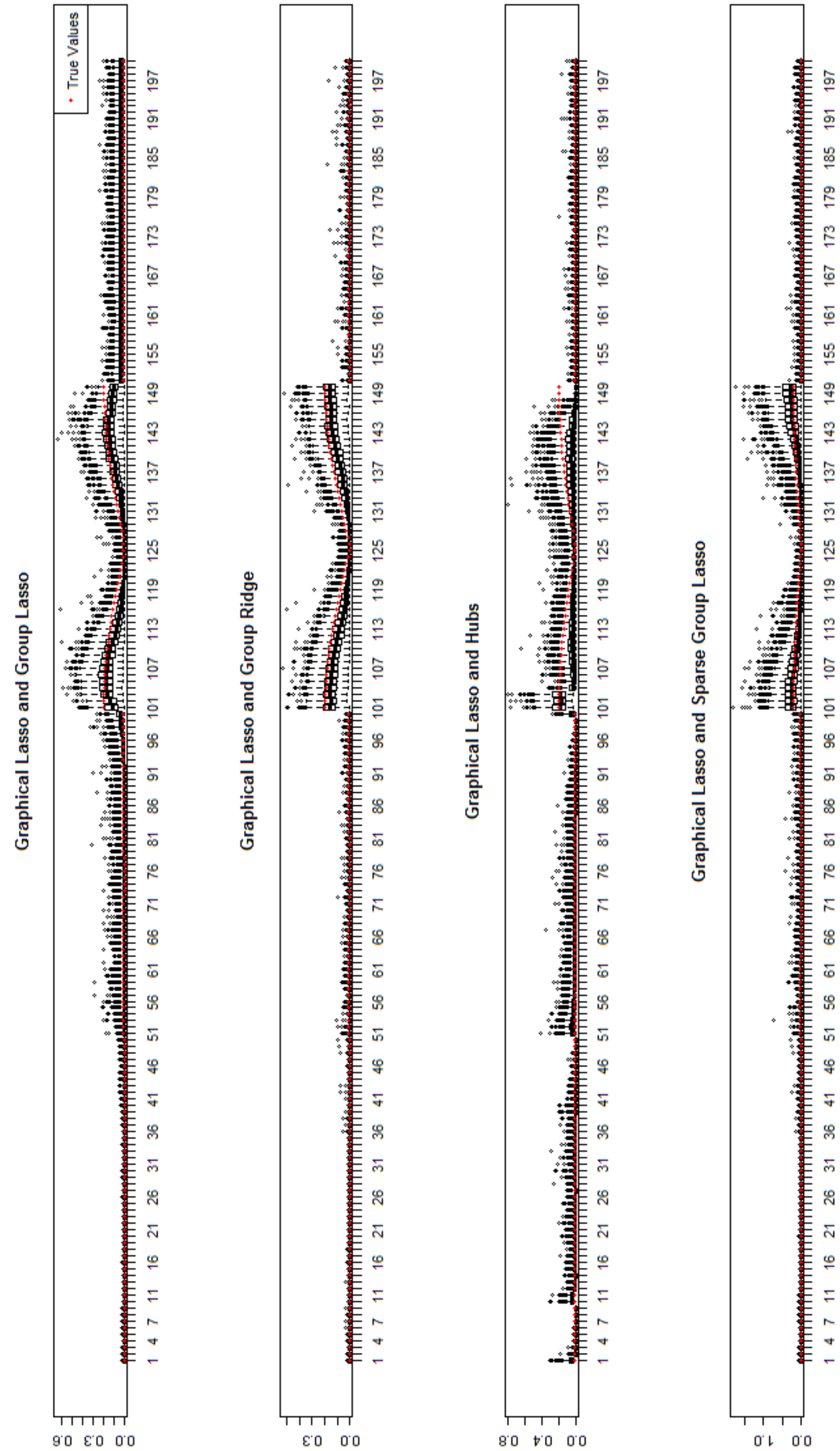

Figure H: Variable selection for scenario b, 4 modules, 200 variables and 50 samples. Box-plots of the absolute values of the estimated parameters for the 200 variables over the 500 datasets simulated are plotted. The red points represent the absolute average true values over the 500 datasets.

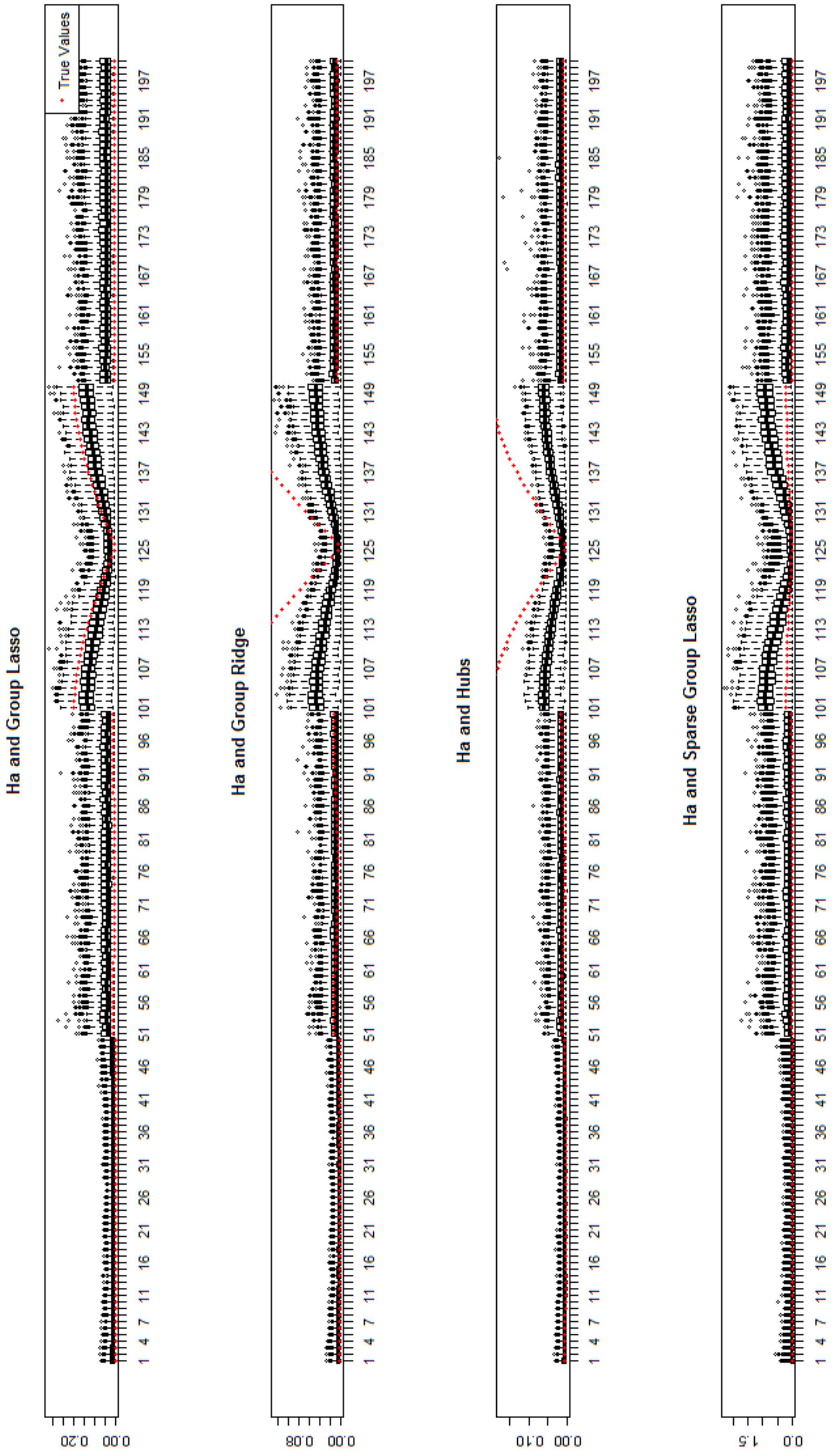

Figure I: Variable selection for scenario b, 4 modules, 200 variables and 50 samples. Box-plots of the absolute values of the estimated parameters for the 200 variables over the 500 datasets simulated are plotted. The red points represent the absolute average true values over the 500 datasets.

2.2 8 modules

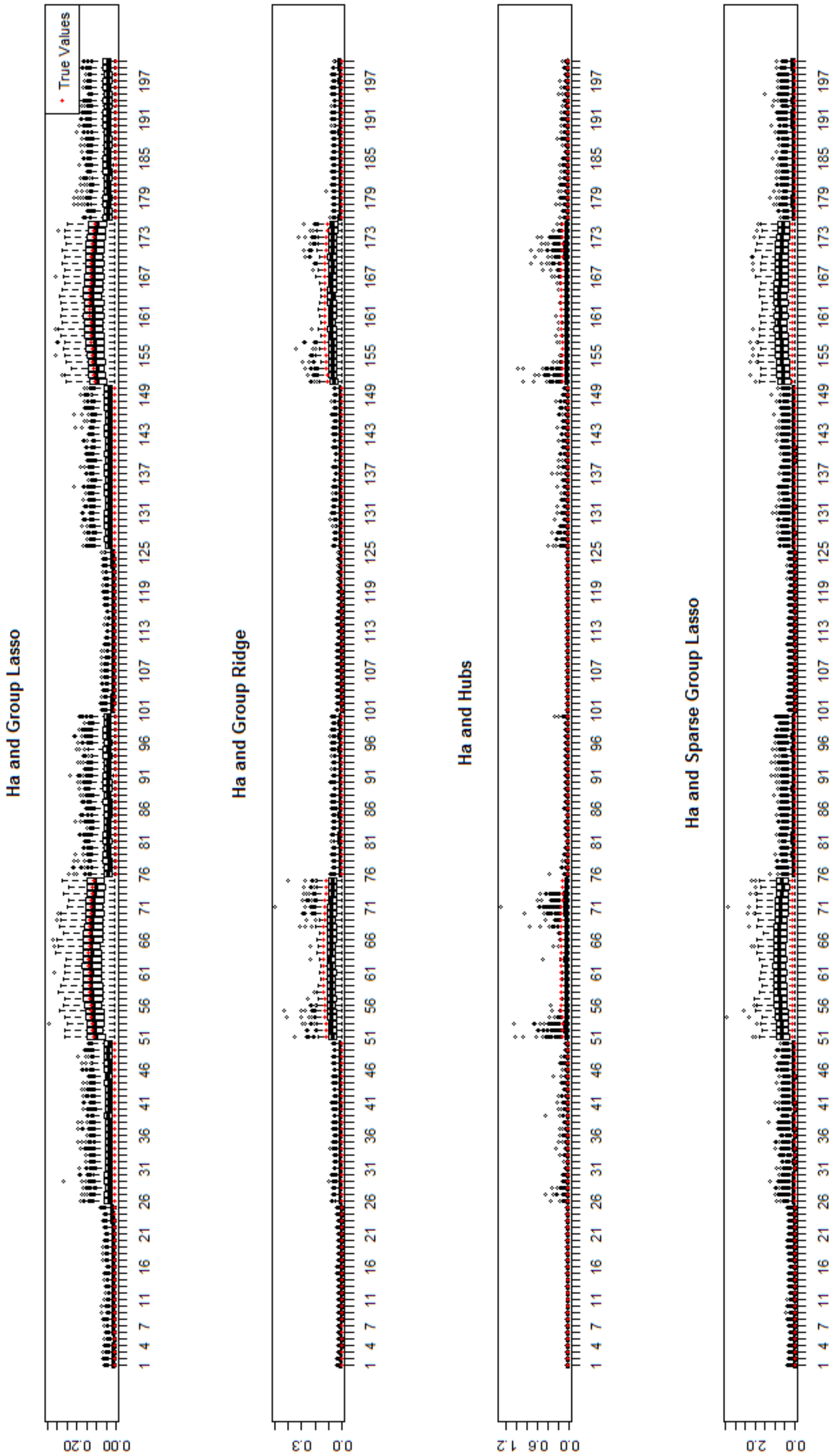

Figure J: Variable selection for scenario b, 8 modules, 200 variables and 50 samples. Box-plots of the absolute values of the estimated parameters for the 200 variables over the 500 datasets simulated are plotted. The red points represent the absolute average true values over the 500 datasets.

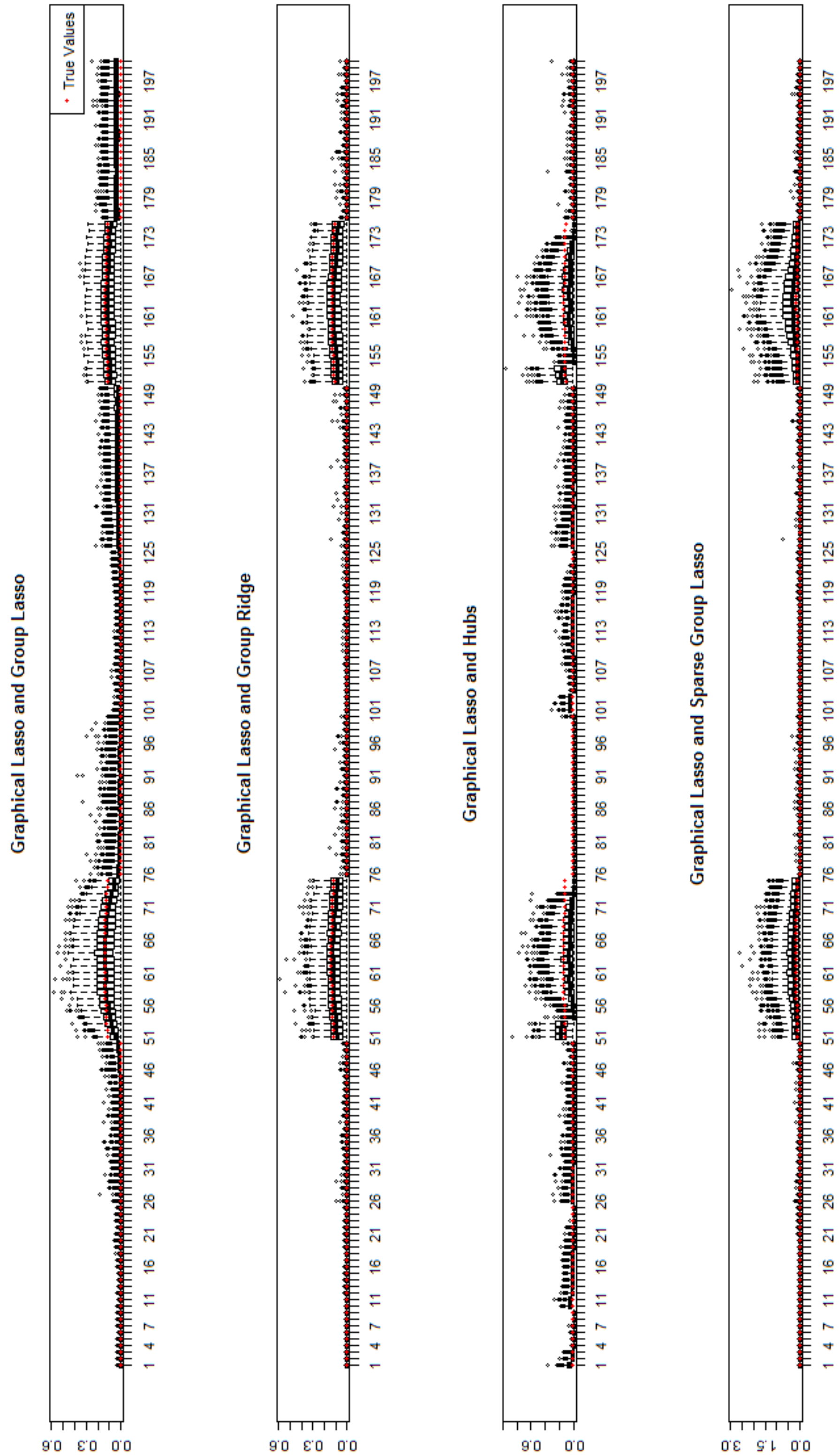

Figure K: Variable selection for scenario b, 8 modules, 200 variables and 50 samples. Box-plots of the absolute values of the estimated parameters for the 200 variables over the 500 datasets simulated are plotted. The red points represent the absolute average true values over the 500 datasets.

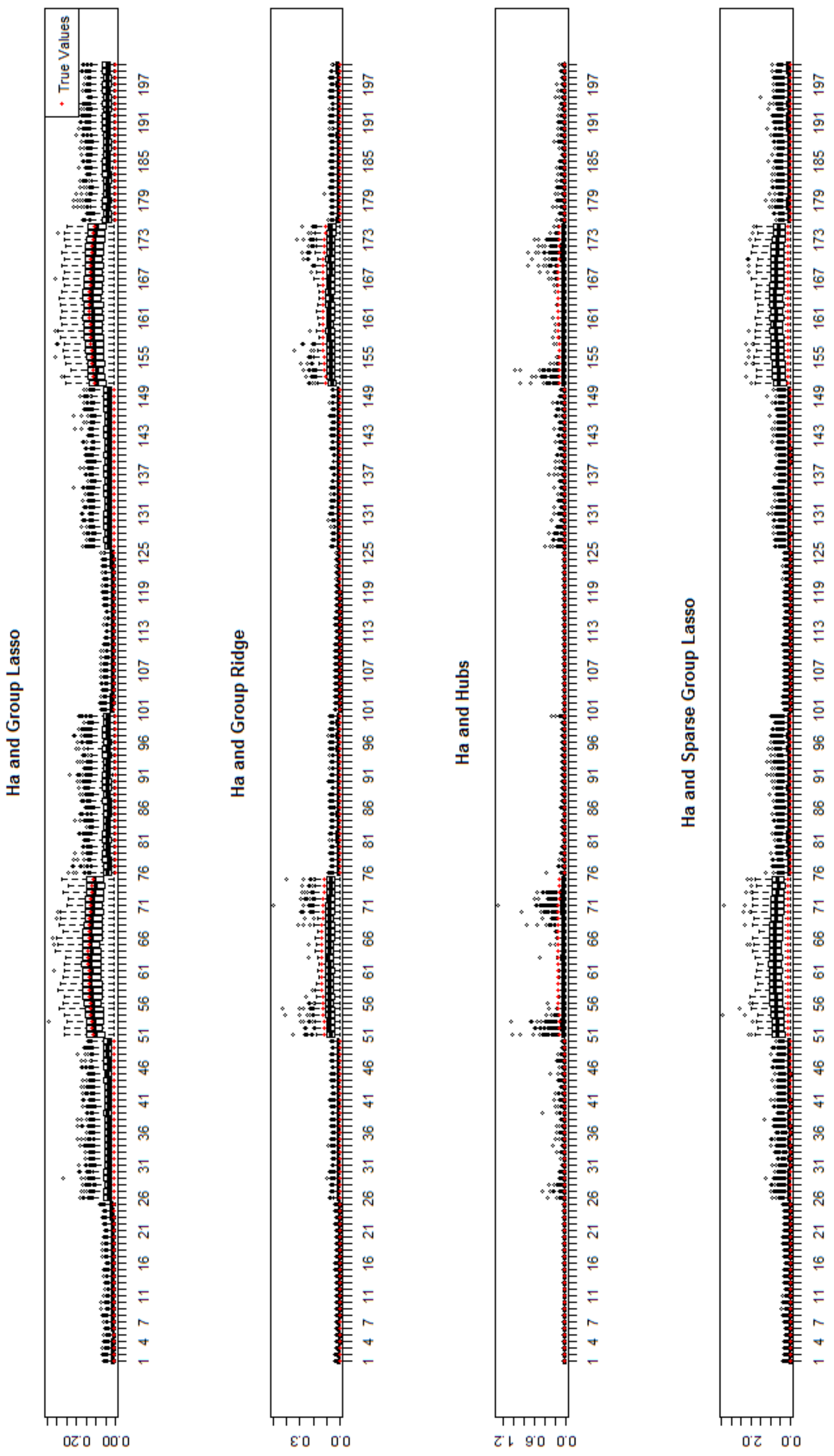

Figure L: Variable selection for scenario b, 8 modules, 200 variables and 50 samples. Box-plots of the absolute values of the estimated parameters for the 200 variables over the 500 datasets simulated are plotted. The red points represent the absolute average true values over the 500 datasets.

3 Scenario c

3.1 4 modules

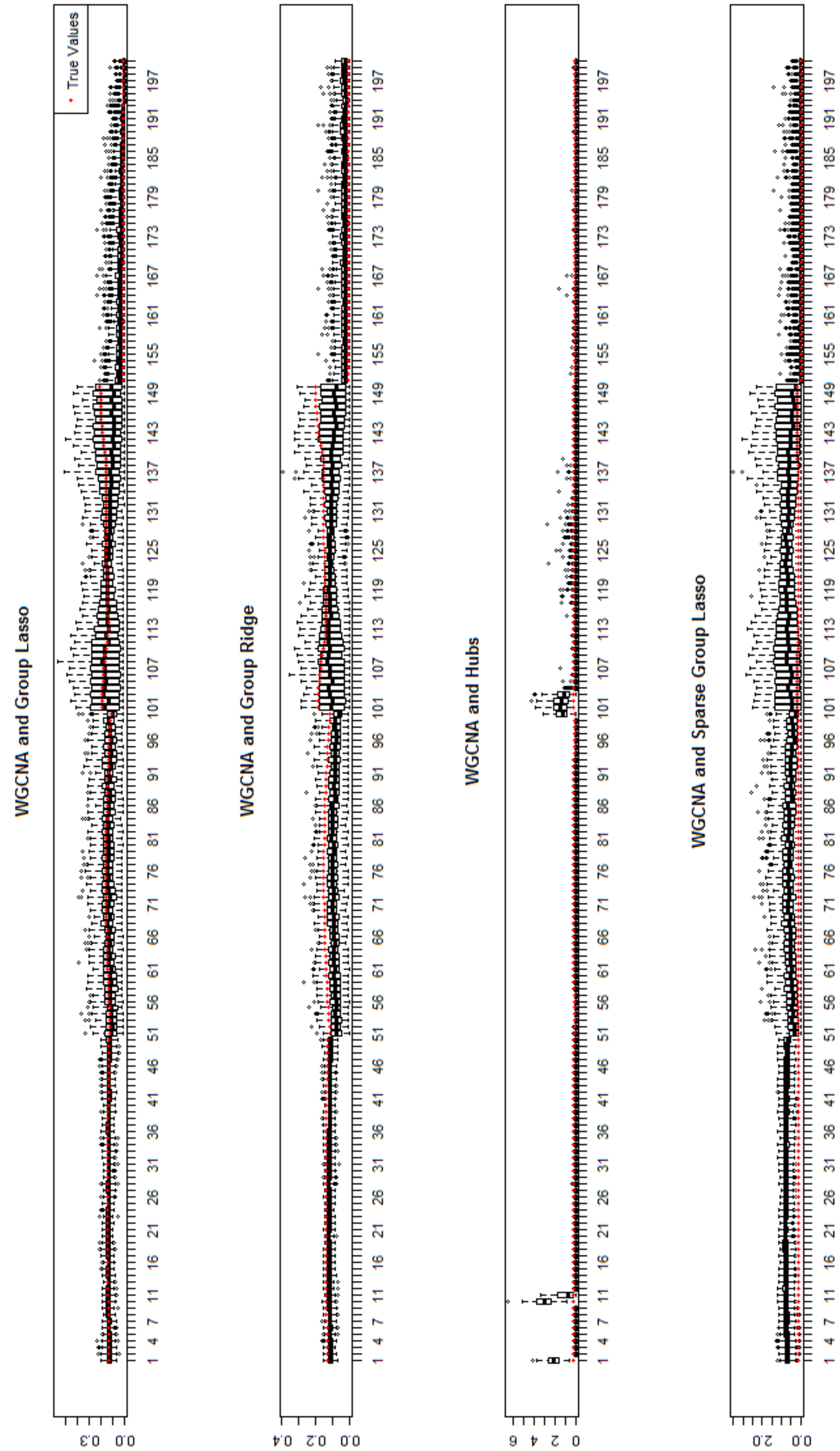

Figure M: Variable selection for scenario c, 4 modules, 200 variables and 50 samples. Box-plots of the absolute values of the estimated parameters for the 200 variables over the 500 datasets simulated are plotted. The red points represent the absolute average true values over the 500 datasets.

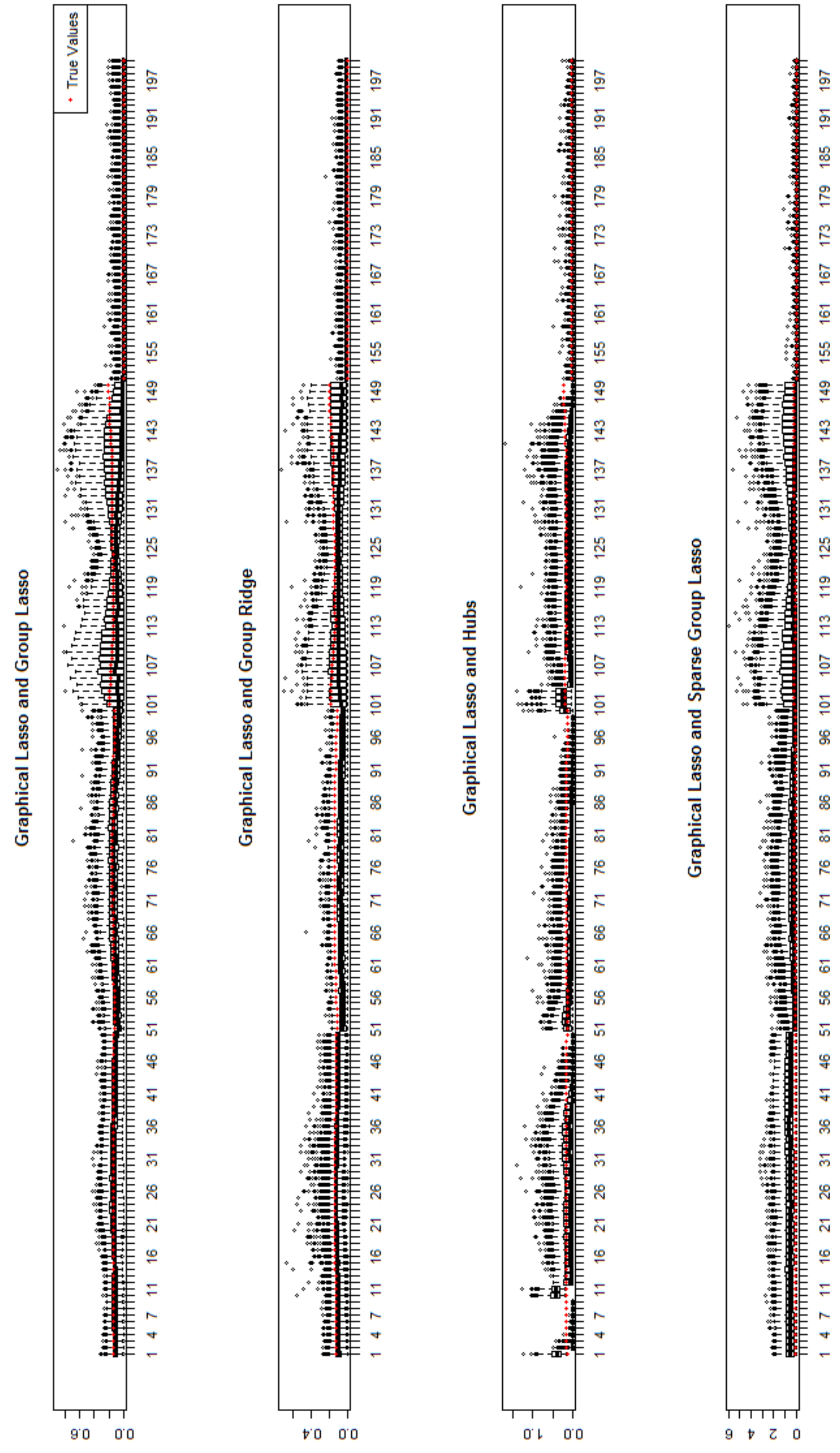

Figure N: Variable selection for scenario c, 4 modules, 200 variables and 50 samples. Box-plots of the absolute values of the estimated parameters for the 200 variables over the 500 datasets simulated are plotted. The red points represent the absolute average true values over the 500 datasets.

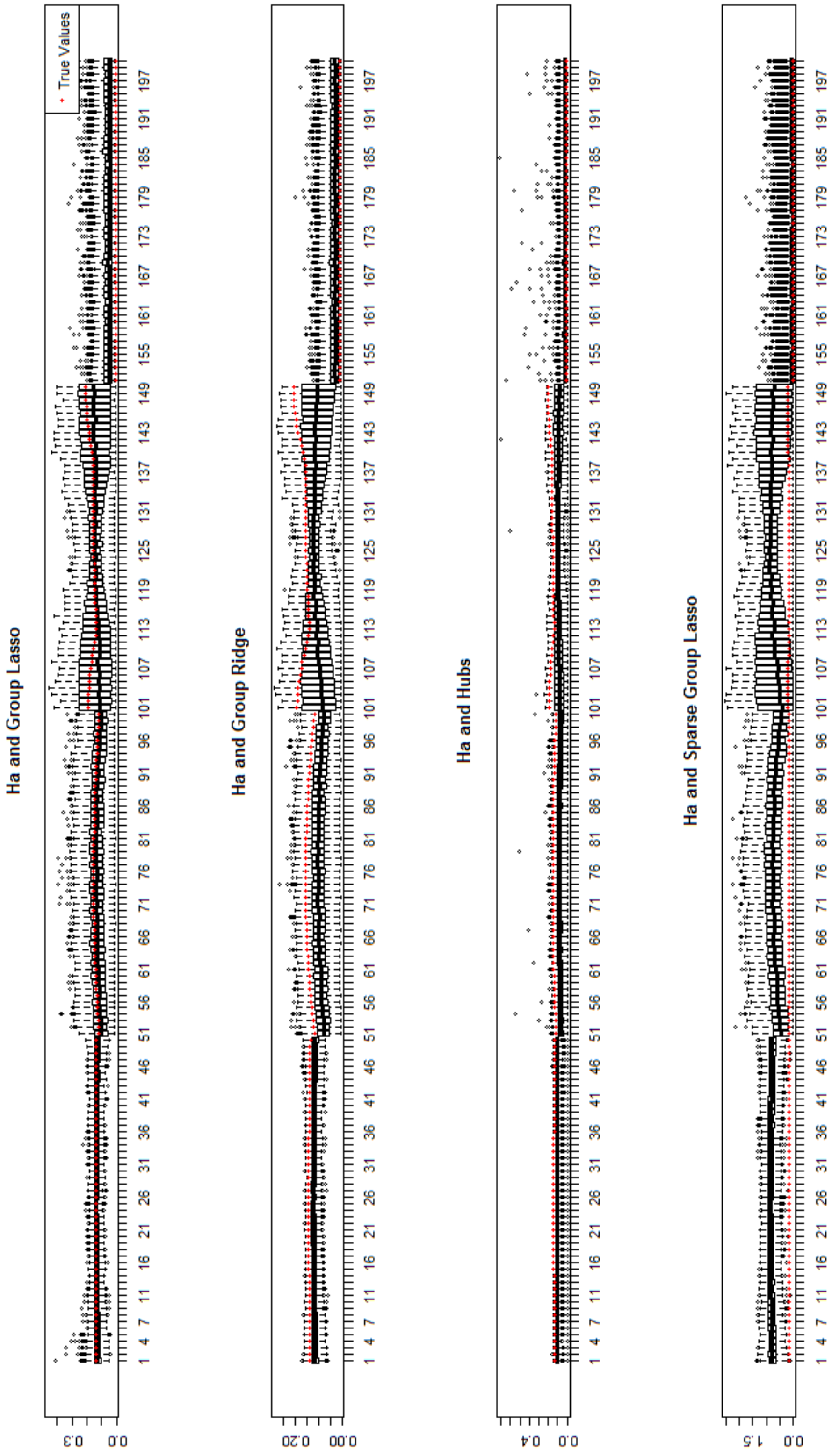

Figure O: Variable selection for scenario c, 4 modules, 200 variables and 50 samples. Box-plots of the absolute values of the estimated parameters for the 200 variables over the 500 datasets simulated are plotted. The red points represent the absolute average true values over the 500 datasets.

3.2 8 modules

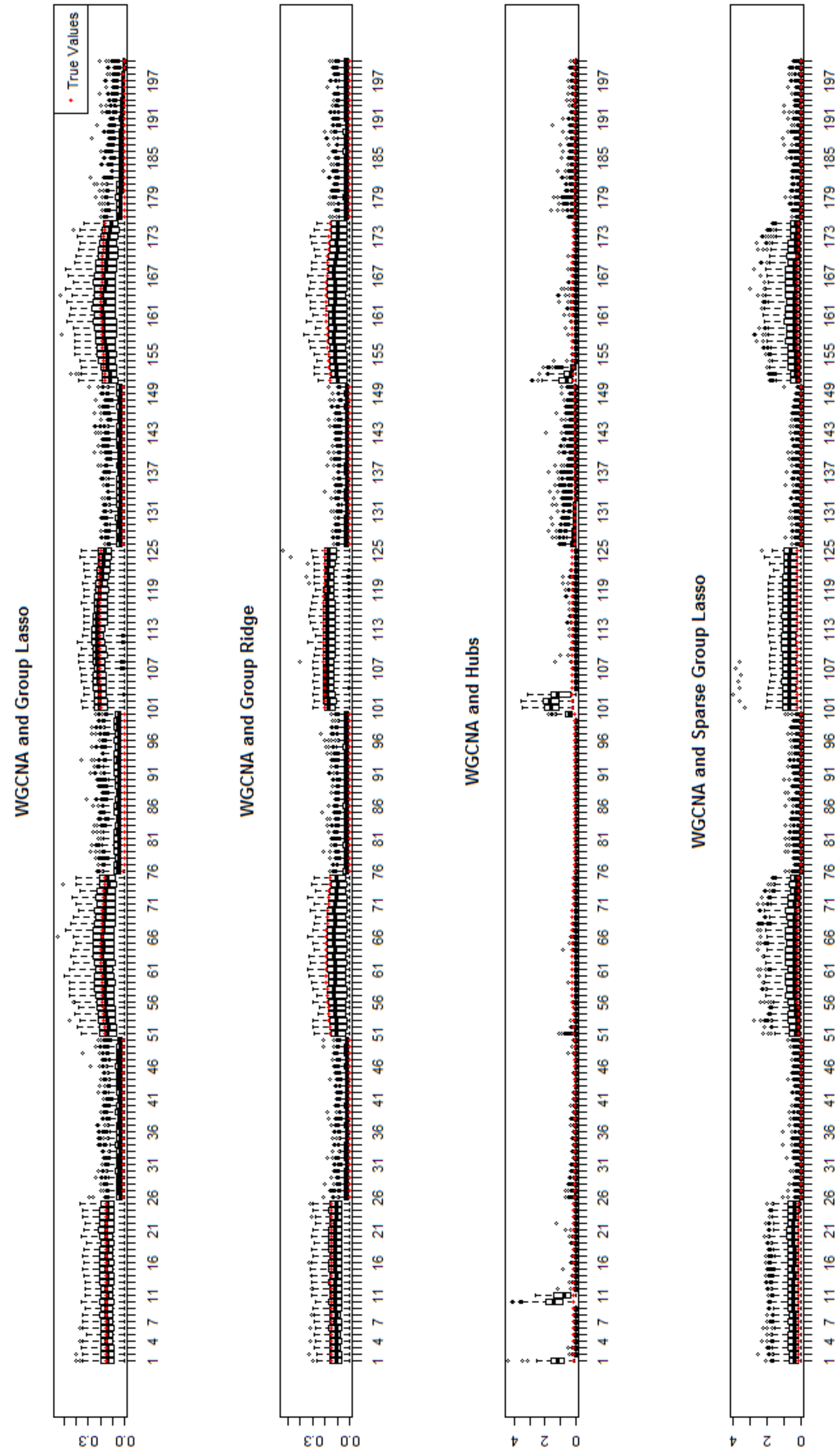

Figure P: Variable selection for scenario c, 8 modules, 200 variables and 50 samples. Box-plots of the absolute values of the estimated parameters for the 200 variables over the 500 datasets simulated are plotted. The red points represent the absolute average true values over the 500 datasets.

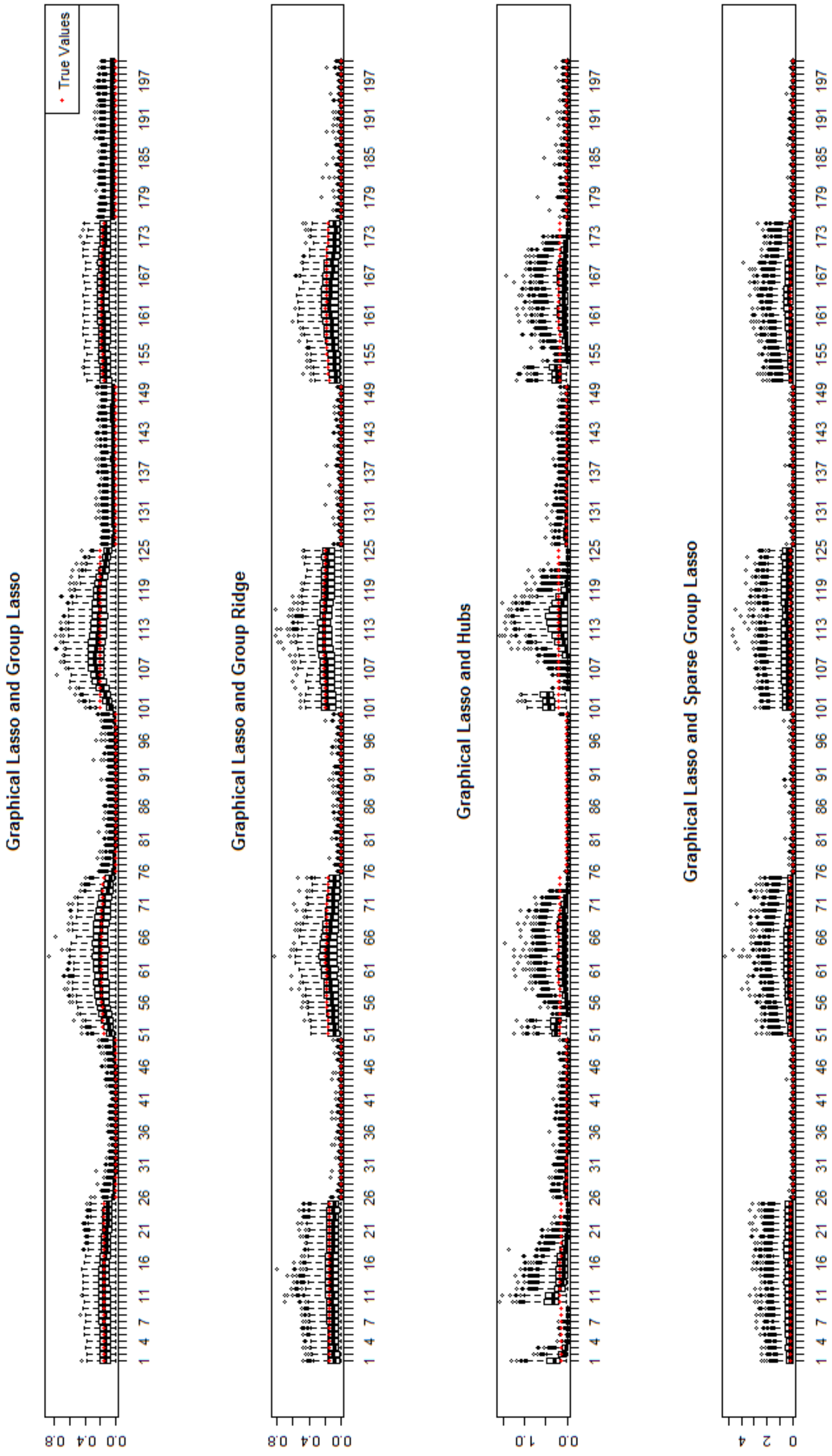

Figure Q: Variable selection for scenario c, 8 modules, 200 variables and 50 samples. Box-plots of the absolute values of the estimated parameters for the 200 variables over the 500 datasets simulated are plotted. The red points represent the absolute average true values over the 500 datasets.

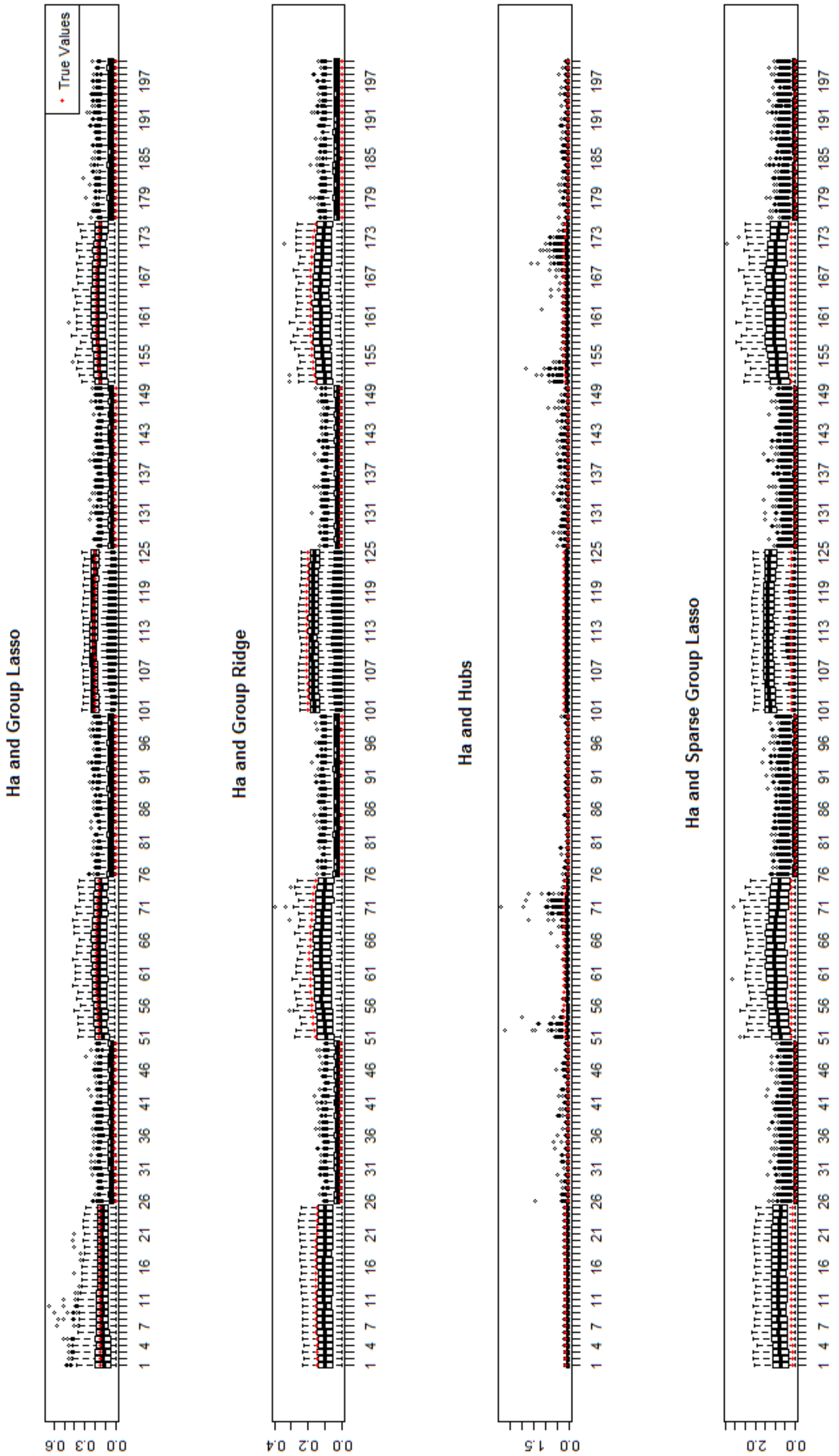

Figure R: Variable selection for scenario c, 8 modules, 200 variables and 50 samples. Box-plots of the absolute values of the estimated parameters for the 200 variables over the 500 datasets simulated are plotted. The red points represent the absolute average true values over the 500 datasets.
