# Supplementary material for: Improving stability of prediction models based on correlated omics data by using network approaches
Source: PLoS One. 2018 Feb 20;13(2):e0192853. doi: 10.1371/journal.pone.0192853 (PMC5819809; doi:10.1371/journal.pone.0192853)
Supplement: S3 File — Detailed description and acronyms of the 57 metabolites studied in the DILGOM study. (PDF) [file pone.0192853.s003.pdf]

S3 File: List of metabolites used in the DILGOM analysis.

Renaud TISSIER, Jeanine HOUWING-DUISTERMAAT, Mar RODRÍGUEZ-GIRONDO

This file contains a detailed description and acronyms of the 57 metabolites studied in the DILGOM study.

Metabolites	Description	Family
ALB	Albumin	Fluid balance
XXLVLDLL	Extremely large VLDL	Total lipid concentrations in lipoprotein subclasses
XLVLDLL	Very large VLDL	Total lipid concentrations in lipoprotein subclasses
LVLDLL	Large VLDL	Total lipid concentrations in lipoprotein subclasses
MVLDLL	Medium VLDL	Total lipid concentrations in lipoprotein subclasses
SVLDLL	Small VLDL	Total lipid concentrations in lipoprotein subclasses
XSVLDLL	Very small VLDL	Total lipid concentrations in lipoprotein subclasses
IDLL	IDL	Total lipid concentrations in lipoprotein subclasses
LLDLL	Large LDL	Total lipid concentrations in lipoprotein subclasses
MLDLL	Medium LDL	Total lipid concentrations in lipoprotein subclasses
SLDLL	Small LDL	Total lipid concentrations in lipoprotein subclasses
XLHDLL	Very large HDL	Total lipid concentrations in lipoprotein subclasses
LHDLL	Large HDL	Total lipid concentrations in lipoprotein subclasses
MHDLL	Medium HDL	Total lipid concentrations in lipoprotein subclasses
SHDLL	Small HDL	Total lipid concentrations in lipoprotein subclasses
IDLC	IDL cholesterol	Cholesterol
LDLC	LDL cholesterol	Cholesterol
HDLC	HDL cholesterol	Cholesterol
SERUMTG	Triglycerides	Lipids
SERUMC	Total cholesterol	Cholesterol
VLDLD	VLDL diameter	Lipoprotein particle size
LDLD	LDL diameter	Lipoprotein particle size
HDLD	HDL diameter	Lipoprotein particle size
HDL2C	HDL2 cholesterol	Cholesterol
APOA1	Apolipoprotein A-I	Apolipoproteins
APOB	Apolipoprotein B	Apolipoproteins

HDL3C	HDL3 cholesterol	Cholesterol
BOHBUT	3-hydroxybutyrate	Ketone bodies
ACE	Acetate	Ketone bodies
ACACE	Acetoacetate	Ketone bodies
ALA	Alanine	Amino acids
CIT	Citrate	Glycolysis-related metabolites
CREA	Creatinine	Fluid balance
GLC	Glucose	Glycolysis-related metabolites
GLN	Glutamine	Amino acids
GLOL	Glycerol	Glycolysis related metabolites
GLY	Glycine	Amino acids
GP	Glycoprotein acetyls	Inflammation
HIS	Histidine	Amino acids
ILE	Isoleucine	Branched-chain amino acids
LAC	Lactate	Glycolysis-related metabolites
LEU	Leucine	Branched-chain amino acids
PHE	Phenylalanine	Aromatic amino acids
PYR	Pyruvate	Glycolysis-related metabolites
TYR	Tyrosine	Aromatic amino acids
UREA	Urea	
VAL	Valine	Branched-chain amino acids
FAW3	Omega-3 fatty acids	Fatty acids
FAW6	Omega-6 fatty acids	Fatty acids
TOTFA	Total fatty acids	Fatty acids
LA	Linoleic acid	Fatty acids
DHA	Docosahexaenoic acid	Fatty acids
MUFA	Monounsaturated fatty acids	Fatty acids
TOTPG	Phosphoglycerides	Lipids
PC	Phosphatidylcholines	Lipids
SM	Sphingomyelins	Lipids
FAW3FA	Omega-3 fatty acids (%)	Fatty acids
FAW6FA	Omega-6 fatty acids (%)	Fatty acids
FALEN	Fatty acid chain length	Saturation measures

Table A: detailed description and acronyms of the 57 metabolites studied in the DILGOM study. HDL and LDL stand for High Density Lipoprotein and Low Density Lipoprotein, respectively.
